# miR-21a-5p Promotes Inflammation following Traumatic Spinal Cord Injury through Upregulation of Neurotoxic Reactive Astrocyte (A1) Polarization by Inhibiting the CNTF/STAT3/Nkrf Pathway

**DOI:** 10.7150/ijbs.60509

**Published:** 2021-07-05

**Authors:** Yining Zhang, Tingting Meng, Jianan Chen, Ying Zhang, Jianning Kang, Xinyu Li, Guilian Yu, Lige Tian, Zhengxin Jin, Hui Dong, Xiaodi Zhang, Bin Ning

**Affiliations:** 1 Central Hospital Affiliated to Shandong First Medical University, Shandong First Medical University & Shandong Academy of Medical Sciences, Jinan, Shandong 250013, China.; 2 Jinan Central Hospital, Cheeloo College of Medicine, Shandong University, No.105, Jiefang Road, Jinan, Shandong 250013, China.; 3 School of Clinical Medicine, Weifang Medical University, Weifang 261053, China.

**Keywords:** microRNA-21a-5p, Traumatic Spinal Cord Injury (TSCI), neurotoxic reactive astrocyte (A1s), ciliary neurotrophic factor (CNTF), ciliary neurotrophic factor receptor α (CNTFR α)

## Abstract

Reactive astrocytes are implicated in traumatic spinal cord injury (TSCI). Interestingly, naïve astrocytes can easily transform into neurotoxic reactive astrocytes (A1s) with inflammatory stimulation. Previous studies demonstrated that microRNA(miR)-21a-5p was up-regulated in spinal cord tissue after TSCI; however, it is not clear whether this affected reactive astrocyte polarization. Here, we aim to detect the effects of miR-21a-5p on the induction of A1 formation and the underlying mechanisms. Our study found that the expression of miR-21a-5p was significantly increased while that of *Cntfr α* was decreased, since naïve astrocytes transformed into A1s 3 days post-TSCI; the binding site between miR-21a-5p and *Cntfr α* was further confirmed in astrocytes. After treatment with CNTF, the levels of A1 markers decreased while that of A2 increased. The expression of A1 markers significantly decreased with the downregulation of miR-21a-5p, while *Cntfr α* siRNA treatment caused the opposite both *in vitro* and *in vivo*. To summarize, miR-21a-5p/Cntfr α promotes A1 induction and might enhance the inflammatory process of TSCI; furthermore, we identified, for the first time, the effect and potential mechanism by which CNTF inhibits naïve astrocytes transformation into A1s. Collectively, our findings demonstrate that targeting miR-21a-5p represents a prospective therapy for promoting the recovery of TSCI.

## Introduction

Traumatic spinal cord injury (TSCI) usually leads to lifelong disability due to the destruction of the spinal cord 1-3, for which there is currently no effective treatment. Previous studies have reported that the total cost of long-term treatment is at least $1.1 million per patient in the United States 4, 5. Several researches reported that neuroinflammation was associated with the weakening of axonal regenerative activity, leading to the poor recovery of injured tissue in the early stages, which might influence the poor prognosis of TSCI 6-9.

Astrocytes are the most abundant resident cells in the central nervous system (CNS), exerting a housekeeping role in healthy CNS 10, 11. However, naïve astrocytes tend to be induced into reactive astrocytes by CNS injury or disease 12. In addition to their neuroprotective effects 13, reactive astrocytes also regulate the neuroinflammatory response to CNS damage 9, 11, 14, 15. Recently, a series of studies have found that reactive astrocytes can be divided into two phenotypes: neurotoxic reactive astrocytes (A1s) and neuroprotective reactive astrocytes (A2s). These studies showed A1s were induced by IL-1α, TNF-α, and C1q (hereafter termed A1 cocktail) secreted by lipopolysaccharide-stimulated M1 microglia 16-19. They lost the original function as astrocytes and increased the secretion of inflammatory cytokines, leading to neurotoxicity 9, 16, 18, 21. On the other hand, A2s were induced by ischemia 16, 17, 20, up-regulated neurotrophic factors and promoted the survival of neurons 20, 22, 23. Markers of A1s include complement component 3 (C3), serpin family G member 1 (Serping1), and histocompatibility 2, D region locus 1(H2-D1). S100 calcium-binding protein A10 (S100a10) 16, 18 is a marker of A2s.

Ciliary neurotrophic factor receptor α (CNTFR α), a receptor of ciliary neurotrophic factor (CNTF) 24, 25, is widely expressed in the CNS 25, 26. By combining with CNTF, CNTFR α activates the JAK/STAT3 signaling pathway 24, 25. First, CNTF was found to be required for axonal regeneration after spinal cord injury (SCI) 25, 27-29. Subsequently, CNTFR α has been found to exert a role in neuroprotection and relieve neuroinflammation by regulating astrogliosis 25, 30-32 and the function of microglia 8, 33-35. Previous studies reported that the NF-κB signaling pathway is essential for A1 polarization 17, 36, 37 while the STAT3 signaling pathway might promote A2 and inhibit A1 transformation 17, 20. Similarly, STAT3 may also be essential for glial scar formation and astrocytic neuroprotection after spinal cord injury (SCI) 13, 32, 38-40. It is worth mentioning that whether CNTF affects the polarization of reactive astrocytes through the STAT3 signaling pathway has not yet been reported.

MicroRNAs, small non-coding RNAs each containing 19-25 nucleotides 41, can downregulate the expression of mRNA by targeting the 3'UTR 41, 42. Recently, several studies have reported that microRNAs might be necessary for recovery from SCI 43, 44. Significantly, upregulation of miR-21a-5p was shown to regulate the formation of glial scars 45 and fibrosis 46, 47 by inhibiting axon regeneration after SCI 48. In addition, miR-21a-5p was also confirmed to promote the polarization of A2s 20 in ischemic spinal cord injury (ISCI). Nevertheless, it remains unclear whether miR-21a-5p plays a key role in regulating the induction of A1s after TSCI.

In this study, we sought to assess the relationship between miR-21a-5p and the polarization of reactive astrocytes, and detect possible mechanisms linked to TSCI. Our study provides a prospective viewpoint of the polarization of reactive astrocytes, offering possible solutions for TSCI repair.

## Materials and methods

### Animals and TSCI model

Male C57BL/6 mice (6-8 weeks old) were purchased from the Pengyue Experimental Animal Breeding Co., Ltd. (Jinan, China). The animals were housed in clean cages under conditions of 22-24 °C, relative humidity of 30-60%, and a 12-h light/dark cycle, with adequate food and water.

Mice were anesthetized with 3% pentobarbital (30 mg/kg, i.p.), after which T8-T10 laminectomy was performed to expose the spinal cord. The TSCI model was generated using a 68099Ⅱ precision percussion device (RWD Life Science, Shenzhen, China; 1 m/s speed, 2 mm depth, 1 s dwell time). The success of the procedure was confirmed by the appearance of tail spasms and retraction-like flutters of the legs. After surgery, mice were kept on a heated pad for 24 h, and bladders were manually voided twice daily.

### Animal experiments

For one experiment, mice were divided into sham and TSCI groups. The spinal cord tissues of sham and 3 days post-TSCI mice were extracted for the gene chip assay to assess the differential expression of mRNA.

In a separate experiment, mice were randomly divided into four groups: sham, negative control (NC), antagomir-21 (a miRNA inhibitor), and antagomir-21+*Cntfr α* siRNA. In the NC or antagomir-21 groups, antagomir-NC or antagomir-21 (2.5 μL, 2 μmol/mL) (RiboBio, Guangzhou, China) was intrathecally injected three times (0, 1, 2) after mice were subjected to TSCI. Scrambled siRNA (1 μL, 0.5 μmol/mL, RiboBio) was intrathecally injected once on day (0) in the NC group. In the antagomir-21+*Cntfr α* siRNA group, after intrathecal injection of antagomir-21, *Cntfr α* siRNA 0.5 nmol (1 μL, 0.5μmol/mL) was injected once (0). In the sham group, only laminectomy was performed.

Three days after TSCI, mice were anesthetized with 3% pentobarbital, and the spinal cord tissues were carefully removed after transcardial perfusion. For staining, the tissues were fixed overnight with 4% paraformaldehyde at 4 °C. After dehydration in xylene and alcohol gradients, the tissues were embedded in paraffin. For the gene chip analysis, qRT-PCR, and western blotting, either the sample was quickly placed in a frozen tube and transferred to liquid nitrogen, or total RNA or protein was immediately extracted.

### mRNA gene chip assays

Spinal cord tissues were extracted from sham and 3d post-TSCI mice and frozen in liquid nitrogen. Gene chip assays were performed by Genechem Co. (Shanghai, China).

### Bioinformatics analysis

PicTar, TargetScan, and miRanDa bioinformatics were used to predict potential target genes of miR-21a-5p.

### Cell culture and transfection

For all procedures, cells were cultured in Dulbecco's modified essential medium (Gibco, China) with 10% fetal bovine serum (FBS, Gibco, Australia) and 1% penicillin-streptomycin (Solarbio, China) in a humidified incubator with 5% CO2 at 37 °C. FBS was heat-treated at 56 °C for 30 min prior to use.

Primary mouse astrocytes were extracted from 1- to 3-day-old C57BL/6 mice; manual dissection of layers of the brain and spinal cord was performed and the meninges and blood vessels were removed. After fragmentation, tissues were digested with trypsin (Solarbio) at 37 °C for 15 min. The cell suspension was centrifuged and re-suspended in the cell culture solution. A 1-h pre-plating step was performed to further remove the fibroblasts. The unattached glia were seeded in a 100-mm dish and the medium was replaced after 48 h of culture. For purifying astrocytes, the culture media was supplemented with cytosine arabinoside (Ara-C, 1 μM, Solarbio). After the cultures reached confluence, they were shaken at 160-180 rpm overnight at 37 °C to remove non-astrocytes, leaving primary mouse astrocytes. Third generation astrocytes were seeded in 6-well plates for further experiments.

Next, miR-21 mimic, inhibitor, or negative control, and/or *Cntfr α* siRNA or scrambled (NC) (RiboBio, Wuhan, China) were transfected to astrocytes for 72 h when they were at 50-70% density. Transfection was performed using Opti-MEM (Gibco) and Lipofectamine®2000 Reagent (Invitrogen, ThermoFisher, Shanghai, China). After pretreatment with CNTF (100 ng/mL, PeproTech) for 24 h, the cells were treated with IL-1α (3 ng/mL, PeproTech), TNF-α (30 ng/mL, PeproTech), and C1q (400 ng/mL, CUSABIO) to induce A1s. RNA or protein was extracted for subsequent use.

### RNA extraction and quantitative real-time polymerase chain reaction (qRT-PCR)

TRIzol^TM^ (Accurate Biology, Hunan, China) was used to extract total RNA from astrocytes or spinal cord tissue. The SpectraMax®QuickDrop^TM^ spectrophotometer was used to detect the concentration of total RNA. Total RNA was stored at -80 °C or immediately used.

The Mir-X^TM^miRNA First-Strand Synthesis Kit (Takara, Dalian, China) and miR-21a-5p primer (Takara) were used to detect the expression level of miR-21a-5p, and U6 was used as an endogenous control to normalize the results. Total RNA (1000 ng) was prepared for reverse transcription using Exo M-MLV RT Kit with gDNA Clean for qPCR II (Accurate Biology). qRT-PCR was performed using the SYBR®Green Premix Pro Taq HS qPCR Kit (Accurate Biology) and a LightCycler®480II Fast Real-Time PCR System (Roche, Switzerland). GAPDH was used as an endogenous control to normalize the results. The results were analyzed using the 2^-ΔΔCT^ method. The primer pairs used are listed in **Table 1**.

### Protein extraction and western blotting

Astrocytes or spinal cord tissue were lysed using RIPA (Solarbio) with 1% phenylmethylsulfonyl fluoride (Beyotime Biotechnology, Shanghai, China) and 1% Phosphatase Inhibitor Cocktail 100X (CWBio, Beijing, China). Protein concentration was measured using a bicinchoninic acid detection kit (Beyotime Biotechnology). The protein was stored at -80 °C or used immediately.

The protein samples were separated by SDS-PAGE (Beyotime Biotechnology) and transferred to polyvinylidene difluoride membranes (Millipore, USA). After blocking in 5% skim milk powder (BioFroxx, Germany) at 25°C for 1 h, membranes were incubated with primary antibodies at 4 °C overnight. The following day, after washing with phosphate-buffered saline (PBS) with 0.2% Tween-20, membranes were incubated with secondary antibodies at room temperature for 1 h. Finally, proteins were detected using the FluorChem M imaging system (ProteinSimple, USA).

### Antibodies for western blotting

Primary antibodies against the following proteins were used: CNTFR α (1:1000, Santa Cruz, USA), Phospho-STAT3 (p-Stat3, 1:2000, Cell Signaling Technology, USA), STAT3 (1:1000, Cell Signaling Technology), iNOS (1:1000, Cell Signaling Technology), complement component 3 (C3, 1:50, Abcam, UK), S100a10 (1:1000, Abcam), and β-actin (1:5000, ZSGB-Bio, Beijing, China). Secondary antibodies were goat anti-rabbit (1:5000, ZSGB-Bio), goat anti-mouse (1:5000, ZSGB-Bio), goat anti-rat IgG (1:5000, ZSGB-Bio).

### Enzyme-linked immunosorbent assay (ELISA)

The concentration of IL-1β released by A1s was detected by an enzyme-linked immunosorbent assay (ELISA) mouse IL-1β Kit (Invitrogen, ThermoFisher Scientific), according to the manufacturer's protocol. The colorimetric optical density (OD) was measured using a SpectraMax®i3x enzyme labeling instrument (Molecular Devices, USA).

### Dual-luciferase reporter assay

HEK 293t cells were seeded in 24-well plates. For the construction of plasmids, *Mus musculus* wild-type (WT) or mutant (MUT) *Cntfr α* 3'UTR fragments were cloned into PmirGLO Dual-Luciferase miRNA Target Expression Vector (BioSune, Jinan, China). Next, the plasmids (miR-21a-5p mimic, inhibitor, and negative control) were transfected into HEK 293t cells. After 48 h, a 1 × PLA cell lysis buffer was added and the cells were shaken at room temperature for 15 min, after which the cell lysate was collected. Altogether, 20 μL of cell lysate was added to each well of a 96-well plate, and 100 μL of luciferase assay reagent II and 100 μL of Stop & Glo®Reagent were added in turn. We completed this experiment using the Promega Dual-Luciferase system (Promega, Madison, WI, USA). The Centro XS^3^ LB 960 (Berthold, Germany) and MikroWin software were used to detect the firefly luciferase and Renilla luciferase activities and their difference were calculated and analyzed.

### RNA pull-down assay

Mouse *Cntfr α* in pcDNA3.1 (+), antisense-MUT-*Cntfr α* in pcDNA3.1 (+), and pcDNA3.1 (+) were linearized with restriction enzymes and then used for *in vitro* transcription with the MEGAscript T7 Kit (Ambion, Thermo Fisher Scientific, Shanghai, China) and biotin 16 UTP (Ambion, Thermo Fisher Scientific, Shanghai, China) for biotin-labeled RNA transcripts. MEGA clear Kits (Ambion, Thermo Fisher Scientific, Shanghai, China) were used for purification *in vitro*. About 3 μg of biotinylated RNA was heated at 90 °C for 5 min, held at room temperature for 30 min, then cooled to 4 °C. RNA was mixed with 1 mg of protein extracted from primary astrocytes, and incubated with shaking at room temperature for 3 h. Streptavidin agarose beads (60 μL; Invitrogen, Thermo Fisher Scientific, Shanghai, China) was added to each reaction and incubated on a rotating shaker at room temperature for 2 h. The beads were retrieved and a qRT-PCR assay was used to detect the expression of miR-21a-5p in pulled-down RNA.

### Immunofluorescence

For astrocytes, 2.4~2.5×10^5^ cells were seeded in a 24-well plate and grown to 50%-70% density. Then, cells were washed with PBS three times, treated with 4% paraformaldehyde (Solarbio) for 15 min, 0.5% Triton X-100 for 10 min, and then blocked with 10% normal goat serum (Solarbio) for 1 h. Next, the cells were incubated with primary antibody at 4 °C overnight. The next day, samples were incubated with secondary antibodies for 30 min at room temperature. Finally, Antifade Mounting Medium with DAPI (Beyotime Biotechnology) was used for sealing.

Spinal cord tissues were sectioned after paraffin embedding. After treatment with an environmentally friendly transparent dewaxing liquid (Solarbio) for deparaffinizing, slices were hydrated in 100%, 95%, 90%, 80%, and 70% ethanol solution in series. Next, sections were heated in citrate buffer for 15 min for antigen retrieval. The staining procedure was the same as that performed in cells.

An upright fluorescence microscope (Olympus, Tokyo, Japan) was used to obtain images.

### Antibodies for Immunofluorescence

The following antibodies were used: mouse anti-GFAP (1:200, Cell Signaling Technology, USA), rat anti-C3 (1:20, Abcam, UK), rabbit anti-S100a10 (1:200, Abcam), Alexa Fluor®488 goat anti-mouse IgG (1:200, Abcam), Alexa Fluor®594 goat anti-rat IgG (1:200, Abcam), and Alexa Fluor®594 goat anti-rabbit IgG (1:200, Abcam).

### Immunohistochemistry

After antigen retrieval (**see Immunofluorescence**), spinal cord sections were incubated in 3% H_2_O_2_. Next, sections were blocked with 3%BSA (BioFroxx) at room temperature for 1 h followed by incubation with rat anti-C3 (1:20, Abcam, UK) primary antibodies at 4 °C overnight. The next day, after washing with PBS three times, the tissues were incubated for 30 min at room temperature with goat anti-rat secondary antibody (1:200, ZSGB-Bio) and 3,3'-diaminobenzidine tetrahydrochloride (DAB, ZSGB-Bio). Next, sections were stained with hematoxylin (Solarbio) and differentiated with alcohol HCl. After rinsing with tap water, the slices were dehydrated in 70%, 80%, 90%, 95%, and 100% ethanol series, and made transparent with environmentally friendly transparent dewaxing liquid (Solarbio). Next, they were sealed with neutral gum and dried. Images were obtained using an upright fluorescence microscope (Olympus, Tokyo, Japan).

### Chromatin immunoprecipitation (ChIP)

A ChIP assay was performed according to the manufacturer's protocols using the Chromatin Extraction Kit (Abcam) and ChIP Kit Magnetic-One Step (Abcam). The chromatin was extracted from astrocytes and sonicated into 200-1000 bp fragments and immunoprecipitated with an anti-p-Stat3 antibodies. The primer sequences of the *Nkrf* promoter were as follows: Forward: 5'-AACCCCTTTCCAAGGACACAG-3'; Reverse: 5'-AGACTCCTGGTAGGGGACTC-3'. The precipitated chromatin was used for qRT-PCR or PCR. The 100-150 bp PCR products were electrophoresed through a 2% agarose gel (Baygene, Beijing, China) with GelRed™ Nucleic Acid Gel Staining solution (Biosharp, China), and visualized under UV illumination with FluorChem M (ProteinSimple, USA).

### Basso Mouse Scale (BMS)

Motor function assay was evaluated by using the Basso Mouse Scale (BMS), a 9-point scoring system, to test the functional recovery after TSCI in mice. Two well-trained and independent observers assessed the hindlimb locomotor function according to the BMS standard. Investigators were blinded to treatments. The final score of each animal was the average of the two observers.

### Hematoxylin and eosin staining (H&E staining)

Mice were anesthetized with 3% pentobarbital, and the spinal cord tissues were carefully removed after transcardial perfusion on 14 d post-surgery. After deparaffinization (**see Immunofluorescence**), spinal cord tissues were washed with distilled water. Then, hematoxylin and eosin staining was performed. Next, sections were dehydrated with ethanol, and made transparent by environmentally friendly transparent dewaxing liquid (Solarbio). Finally, sections were sealed with neutral gum and dried. Images were obtained using an upright fluorescence microscope (Olympus, Tokyo, Japan).

### Statistical analysis

GraphPad Prism v8.0 software (La Jolla, CA, USA) and SPSS v22.0 software (IBM, Chicago, IL, USA) was used for statistical analysis. Researchers who performed statistical analysis were blinded to treatments. Differences between two groups were analyzed by a Student's t-test. Data were presented as mean ± standard deviation (SD). A one-way analysis of variance (ANOVA) with Bonferroni correction were performed for comparisons between multiple groups. p < 0.05 reflected statistical significance.

## Results

### miRNA and mRNA expression in A1 reactive astrocytes induced by TSCI

**Figure 1:**
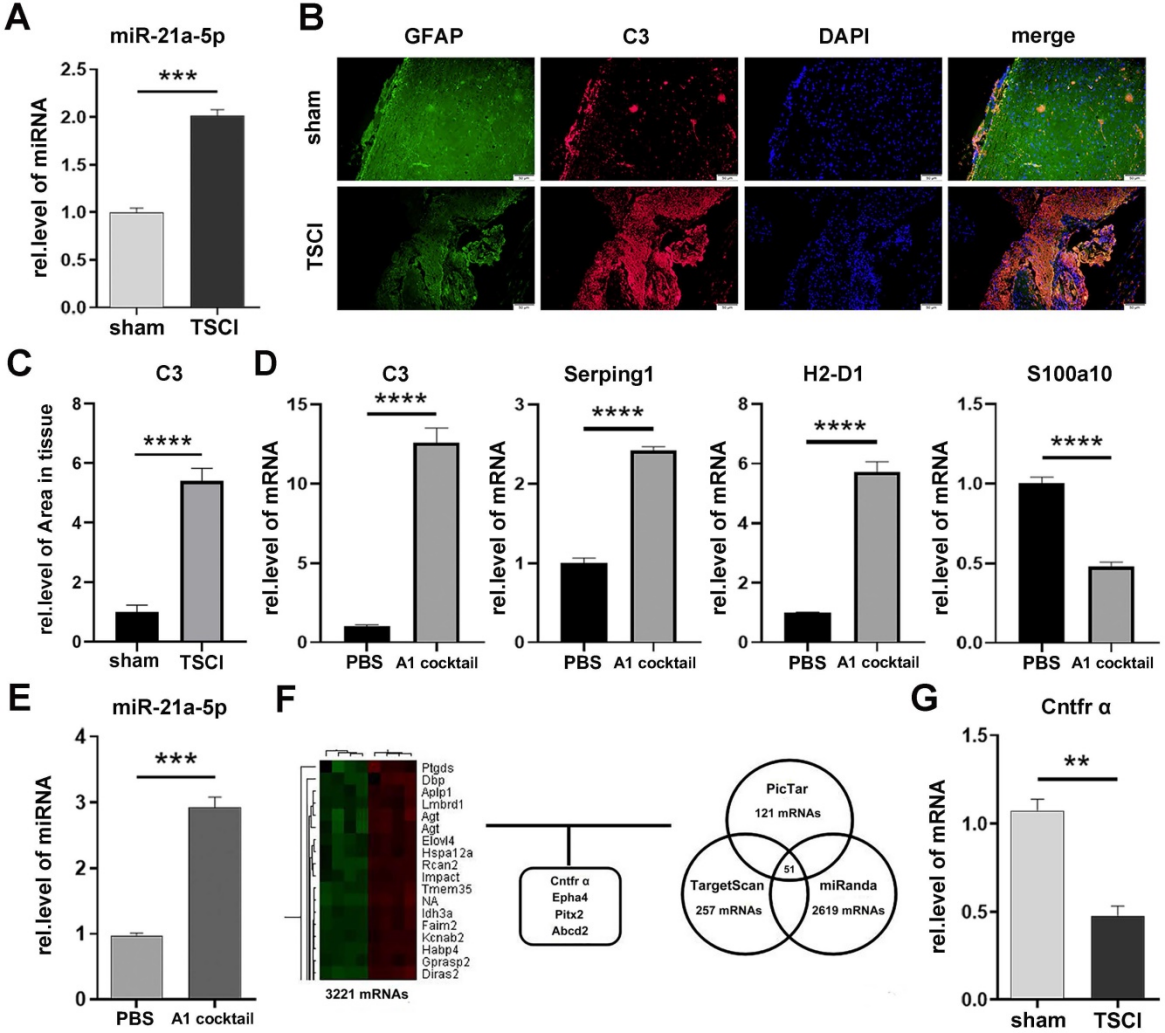
** Neurotoxic astrocytes (A1) appeared 3 days after Traumatic Spinal Cord Injury, alongside increased miR-21a-5p and decreased Cntfr α.** (**A**). miR-21a-5p expression at 3 d post-TSCI was quantified by qRT-PCR. (**B**) The expressions of GFAP (green) and C3 (red) in the sham operation and 3 d post-TSCI groups were detected by immunofluorescence, and the nuclei were stained with DAPI (blue). Scale bar, 50 µm, n=6. Results were analyzed with Image J, GraphPad, and SPSS (**C**). (**D**) qRT-PCR was used to detect the mRNA expression of C3, Serping1, H2D1, S100a10 in cultured astrocytes; GAPDH was used for normalization. (**E**) qRT-PCR was used to detect the expression of miR-21a-5p in cultured astrocytes, U6 was used for normalization. (**F**) Heat map of mRNA that significantly changed in the 3 d post-TSCI group and bioinformatics analysis for choosing the target genes of miR-21a-5p. (**G**) qRT-PCR was used to detect the expression of Cntfr α between the sham group and 3 d post-TSCI group; GAPDH was used for normalization. Results were analyzed with GraphPad and SPSS. The data are expressed as mean ±SD, n=3. *p <0.05, **p <0.01, ***p <0.001, ****p <0.0001.

To assess the expression of miR-21a-5p after TSCI, we performed a qRT-PCR analysis between the sham operation and 3-day post-TSCI groups. The expression of miR-21a-5p after TSCI was significantly increased compared to that in the sham group (Fig. 1A). Immunofluorescence staining revealed the increased expression of C3, an A1s marker, in GFAP^+^ cells after TSCI (Fig. 1B-C), indicating an increase in A1 reactive astrocytes. The significant increase of A1s couldn't be observed on the other stage of TSCI. Thus, we suggest that 3 d post-TSCI is the best stage of assessing A1s markers. Subsequently, to verify the effect of IL-1α (3 ng/mL), TNF-α (30 ng/mL), and C1q (400 ng/mL) on inducing A1s in cultured astrocytes, qRT-PCR was used to detect the expression of mRNA. As shown in Fig. 1D, the expression of *C3*, *Serping1*, and *H2-d1* were upregulated and that of *S100a10* was downregulated in the A1 cocktail group. Meanwhile, the expression of miR-21a-5p was also upregulated in astrocytes treated with A1 cocktail (Fig. 1E), which indicated that miR-21a-5p may affect the induction of A1s.

To verify the target gene of miR-21a-5p, a gene chip assay was performed between the 3 d post-TSCI and sham operation groups, and 1213 downregulated mRNAs identified in the TSCI group were compared with the sham group. All differentially expressed mRNAs showed a >2.0-fold-change threshold (p<0.05). Furthermore, bioinformatics analysis using PicTar, TargetScan, and miRanDa indicated that *Cntfr α*, *Epha4*, *Pitx2*, or *Abcd2* might bind miR-21a-5p at the AUAAGCU binding sequence (Fig. 1F, Supplementary Fig. S1A-D). qRT-PCR further demonstrated that the mRNA levels of *Cntfr α*, *Epha4*, and *Pitx2* decreased at 3 d post-TSCI (Fig. 1G, Supplementary Fig. S2A-C), suggesting that they may be the target genes of miR-21a-5p.

### miR-21a-5p decreased the expression of *Cntfr α* by targeting its 3'UTR in astrocytes

**Figure 2:**
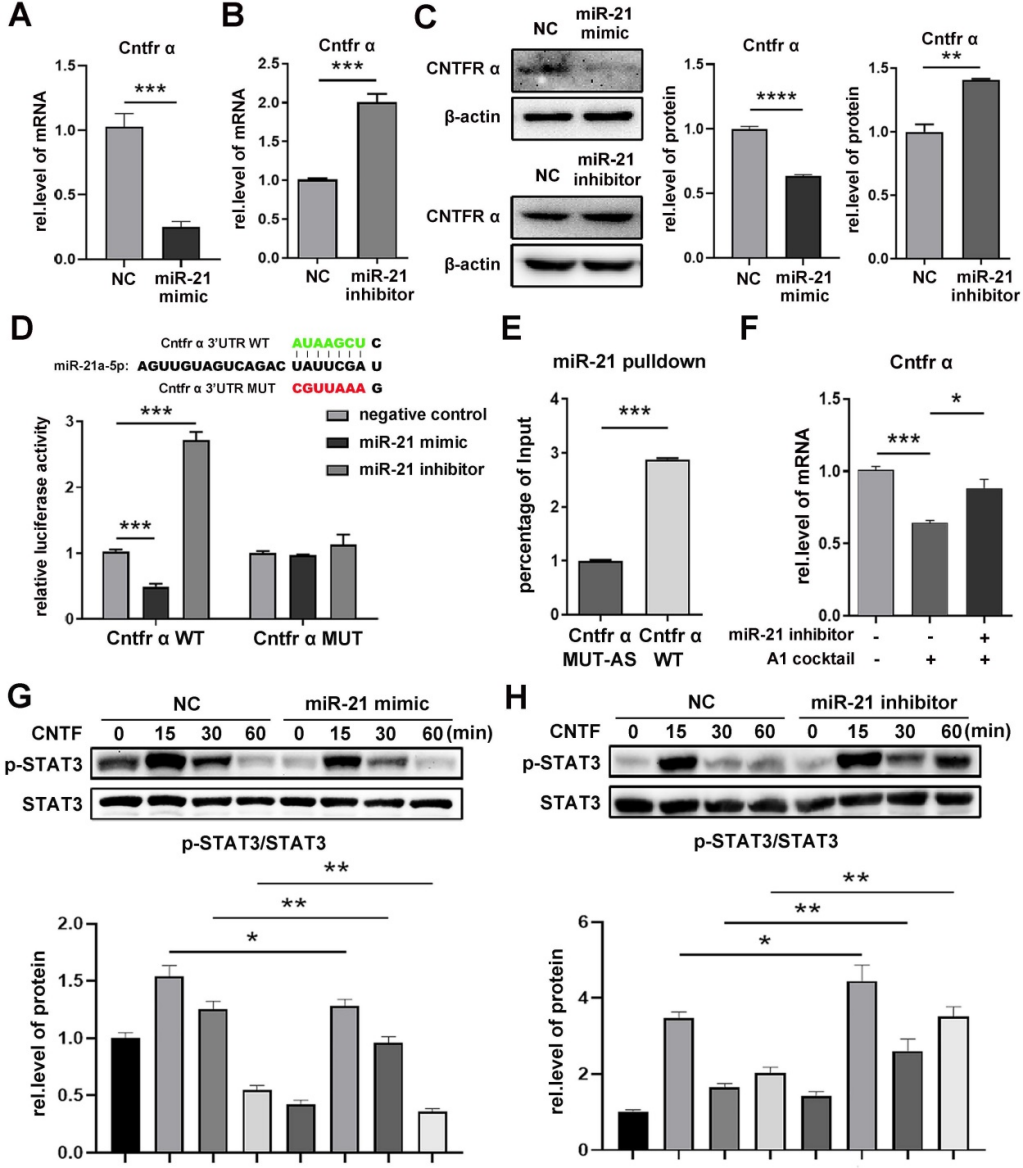
** miR-21a-5p downregulates the expression of Cntfr α by targeting the 3'UTR.** miR-21a-5p mimic, inhibitor, and negative controls were transfected into astrocytes. (**A, B**) qRT-PCR was used to detect the mRNA level of *Cntfr α*, normalized by GAPDH. (**C**) Western blotting was used to detect the protein level of CNTFR α, normalized by β-actin; the results were analyzed by Image J, GraphPad, and SPSS. (**D**) Prediction of targeting sequence between miR-21a-5p and Cntfr α; Dual-luciferase reporter assays were performed to determine the targeting sequence of miR-21a-5p and Cntfr α. (**E**) An astrocyte lysate used for RNA pulldown assay, after which the expression of miR-21a-5p was assessed by qRT-PCR; relative levels of miR-21a-5p were normalized by Input. (**F**) miR-21 inhibitor was used to down-regulate the expression of miR-21a-5p in astrocytes, after which naïve astrocytes were induced into A1s. The expression of Cntfr α was detected by qRT-PCR and normalized by GAPDH. (**G, H**) Astrocytes were transfected with miR-21a-5p mimic, inhibitor, and negative control, then treated with CNTF for 0, 15 30, 60 minutes; Western blotting was used to detect the expression of p-STAT3, STAT3, and β-actin. The results were analyzed with Image J, GraphPad, and SPSS. The data are expressed as mean ±SD, n=3. *p <0.05, **p <0.01, ***p <0.001, ****p <0.0001.

In order to further confirm the target of miR-21a-5p, astrocytes were treated with a miR-21a-5p mimic, inhibitor, or negative control. Next, qRT-PCR was used to determine the mRNA expression in astrocytes. Results showed that miR-21a-5p overexpression significantly decreased the expression of *Cntfr α* and *Epha4* (Fig. 2A, Supplementary Fig. S3A), but did not affect *Pitx2* (Supplementary Fig. S3B). Meanwhile, the levels of *Cntfr α* and *Epha4* were significantly increased after down-regulating miR-21a-5p (Fig. 2B, Supplementary Fig. S3C). Moreover, western blotting also showed similar results on Cntfr α protein expression (Fig. 2C). Here, we confirmed that miR-21a-5p might regulate the polarization of A1s by targeting *Cntfr α*, as the expression of *Cntfr α* was markedly decreased in A1s reactive astrocytes (Supplementary Fig. S3D), which was different from *Epha4* (Supplementary Fig. S3E).

A dual-luciferase reporter assay was performed to verify the binding of miR-21a-5p and *Cntfr α*. The luciferase activity of *Cntfr α*-WT was decreased in the miR-21a-5p mimic group but increased in the miR-21a-5p inhibitor group. However, there was no statistical difference in the luciferase activities of *Cntfr α*-MUT between the miR-21a-5p mimic and inhibitor groups (Fig. 2D). Next, an RNA pulldown assay was used to further assess the binding condition of *Cntfr α* to miR-21a-5p in astrocytes (Fig. 2E). In summary, miR-21a-5p decreased *Cntfr α* expression by targeting the 3'UTR.

Subsequently, to verify that miR-21a-5p regulates inflammation through *Cntfr α*, miR-21a-5p inhibitor was transfected into A1s. Results showed that the expression of *Cntfr α* was enhanced in the miR-21a-5p inhibitor group compared to the control group (Fig. 2F).

Cntfr α is a specific receptor of ciliary neurotrophic factor (CNTF), which can activate the STAT3 signaling pathway. To confirm the influence of miR-21a-5p on the activation of the CNTF/STAT3 pathway, a miR-21a-5p mimic and inhibitor were transfected into astrocytes, then treated with CNTF. Western blotting showed that the phosphorylation of STAT3 was weakened in the miR-21a-5p overexpression group (Fig. 2G) but enhanced in the miR-21a-5p knockdown group (Fig. 2H).

Our results suggest that miR-21a-5p weakens the function of CNTF by targeting the *Cntfr α* 3'UTR.

### CNTF inhibited the induction of A1s by promoting the STAT3/Nkrf pathway *in vitro*

**Figure 3:**
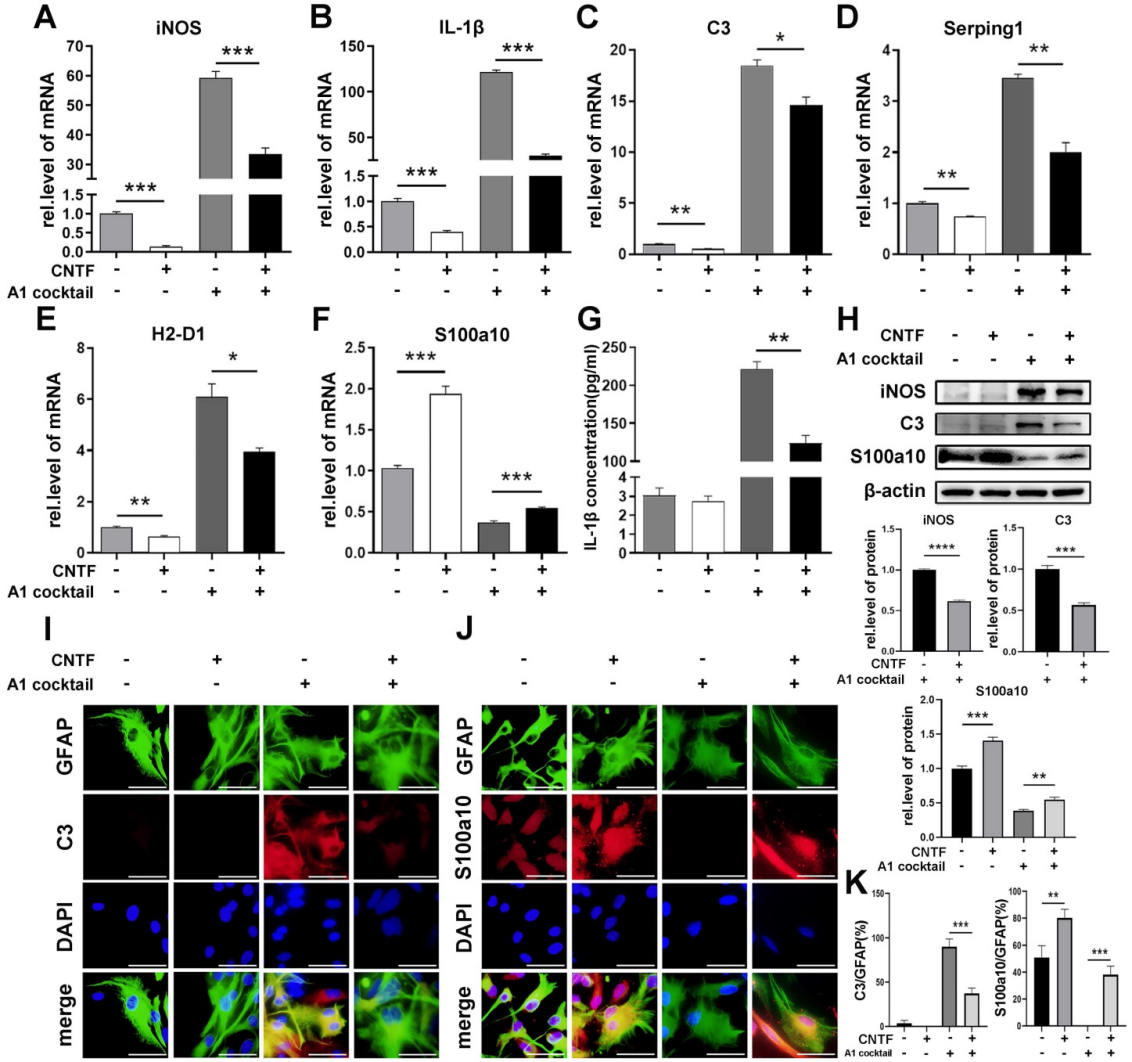
** CNTF down-regulates the polarization of neurotoxic astrocytes (A1).** After pre-treating with CNTF for 24 h, naïve astrocytes were induced into A1 reactive astrocytes by IL-1α, TNF-α, and C1q. PBS was used in the untreated group. (**A-F**) The mRNA expressions of *iNOS, IL-1β, C3, Serping1, H2D1,* and* S100a10* were detected by qRT-PCR, and GAPDH was used for normalization. (**G**) The concentration of IL-1β released by astrocytes was detected by ELISA. The results were analyzed by GraphPad and SPSS. The data are expressed as mean ±SD, n=3. *p <0.05, **p <0.01, ***p <0.001, ****p <0.0001. (**H**) iNOS, C3, S100a10, and β-actin were detected by western blotting, n=3, and results were analyzed with Image J, GraphPad, and SPSS. (**I-J**) Immunofluorescence was used to detect GFAP (green; **I-J**), C3 (red; **I**), S100a10 (red; **J**) and DAPI (blue; nuclei). Scale bar, 20 µm. n=3, the results were analyzed by Image J, GraphPad, and SPSS (**K**). *p <0.05, **p <0.01, ***p <0.001, ****p <0.0001.

To confirm the effect of CNTF/CNTFR α on the polarization of reactive astrocytes, primary astrocytes were pre-treated with CNTF for 24 h. The results of qRT-PCR, western blotting, and the ELISA assay showed that CNTF significantly decreased iNOS and IL-1β expression in A1s (Fig. 3A, G, H). Moreover, CNTF reduced *C3*, *Serping1*, and *H2-D1* expression but upregulated *S100a10* expression in A1s (Fig. 3C-F). Western blotting showed the same results for C3 and S100a10 protein levels (Fig. 3H). Immunofluorescence staining showed high C3 expression and low S100a10 expression in GFAP^+^ A1, which was reversed by CNTF (Fig. 3I-K). Furthermore, naïve astrocytes treated with CNTF exhibited slightly lower A1s marker expression and higher A2s marker expression. These data confirmed that CNTF could exert an anti-inflammatory effect by inhibiting the induction of A1s.

**Figure 4:**
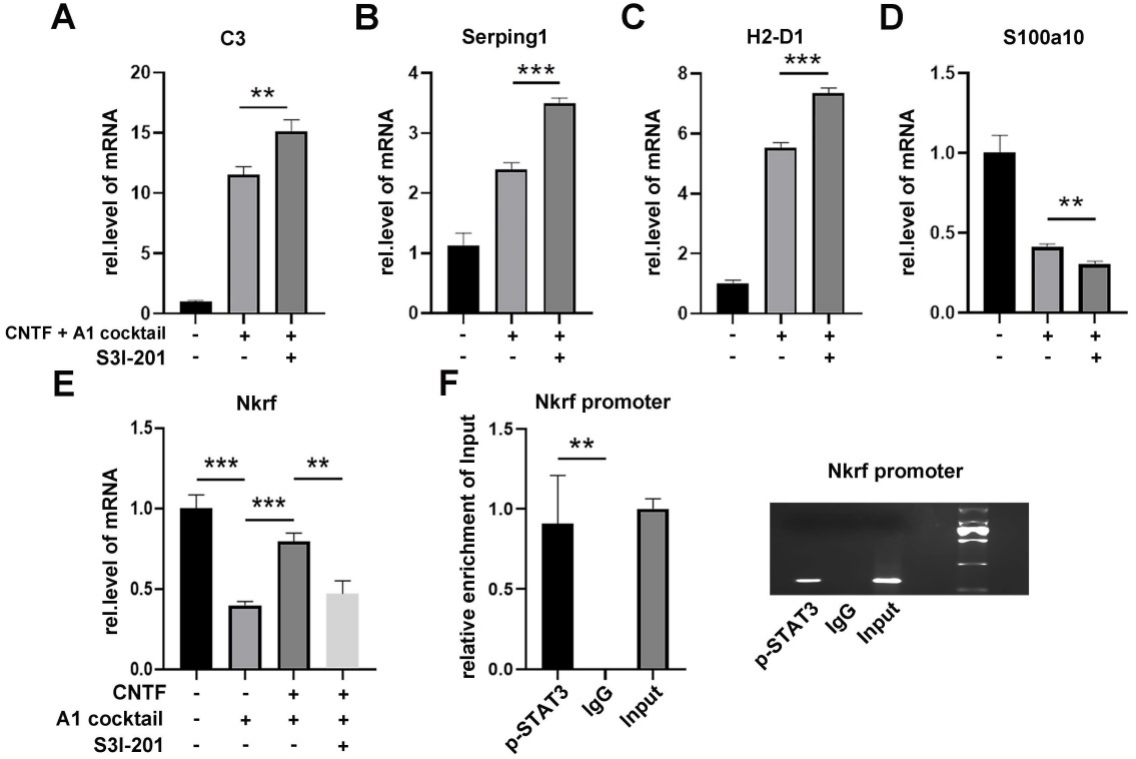
** CNTF downregulates A1s by promoting Nkrf through the STAT3 signaling pathway.** (**A-D**) Astrocytes were treated with S3I-201 or DMSO (untreated) for 1 hour. After pre-treatment with CNTF, astrocytes were induced into A1s. The expression levels of *C3, Serping1, H2D1,* and* S100a10* mRNA were detected by qRT-PCR. (**E**) The expression of *Nkrf* mRNA was detected by qRT-PCR, normalized by GAPDH. (**F**) The targeted binding between p-STAT3 and *Nkrf* promoters was detected by ChIP assay, and Input was used for normalization. The results were analyzed by GraphPad and SPSS. The data were expressed in terms of mean ±SD, n=3. *p <0.05, **p <0.01, ***p <0.001, ****p <0.0001.

Since CNTF activates the STAT3 signaling pathway, S3I-201 (10 μM) was used to repress the activation of the STAT3 pathway (DMSO used as a control group). After pre-treatment with S3I-201, astrocytes were treated with CNTF and A1 cocktail. qRT-PCR showed that expression of *C3*, *Serping1*, and *H2-D1* were increased while* S100a10* was decreased (Fig. 4A-D). These results suggested that CNTF inhibited the induction of A1s through the STAT3 signaling pathway. However, how transcription factor STAT3 plays a role in A1s induction remained unclear.

The NF-kB signaling pathway plays an important role in the induction of A1s. Since NF-κB repressing factor (Nkrf) can effectively inhibit the NF-kB pathway, we speculated that Nkrf might affect the induction of A1s. As shown in Fig. 4E, pre-treating with CNTF significantly enhanced *Nkrf* expression in A1s; this effect was limited in the S3I-201 treatment group. We then determined whether *Nkrf* expression could be promoted by the transcription factor STAT3. The ChIP assay further confirmed that the *Nkrf* promoter was significantly enriched in p-STAT3, which demonstrated the important role of STAT3 in *Nkrf* expression (Fig. 4F).

In conclusion, these data strongly confirmed that CNTF inhibits the induction of A1s through the STAT3/Nkrf pathway.

### miR-21a-5p promoted A1 induction by suppressing the effect of CNTF *in vitro*

**Figure 5:**
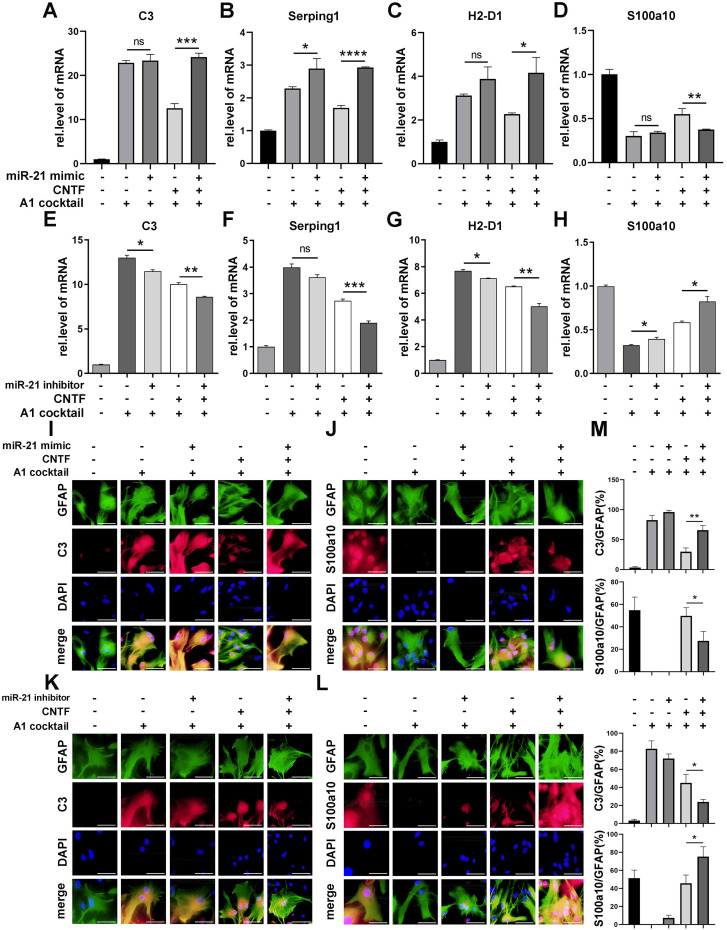
** miR-21a-5p downregulates the inhibitory effect of CNTF on neurotoxic astrocytes (A1).** miR-21 mimic, inhibitor, and negative control were transfected into astrocytes. After pre-treating with CNTF, naïve astrocytes were induced into A1s. (**A-H**) qRT-PCR was used to detect the expression of *C3, Serping1, H2D1,* and* S100a10* mRNA, normalized to GAPDH. Results were analyzed with GraphPad and SPSS. The data are expressed as mean ±SD, n=3. *p <0.05, **p <0.01, ***p <0.001, ****p <0.0001. (**I-L**) Astrocytes were detected by immunofluorescence staining for GFAP (green; **I-L**), C3 (red; **I, K**), S100a10 (red; **J, L**), and DAPI (blue; nuclei). Scale bar, 20 µm; n=3; the results were analyzed with Image J, GraphPad, and SPSS (**M**). *p <0.05, **p <0.01, ***p <0.001, ****p <0.0001.

To verify whether miR-21a-5p affected the function of CNTF, a miR-21 mimic and inhibitor were transfected into astrocytes, that were pre-treated with CNTF. The inhibitory effect of CNTF on A1s induction was significantly weakened after treatment with the miR-21a-5p mimic (Fig. 5A-D). Meanwhile, the effect of CNTF was enhanced by transfection with the miR-21a-5p inhibitor (Fig. 5E-H). Immunofluorescence staining showed that the effect of CNTF on decreasing C3 expression and increasing S100a10 expression in GFAP^+^ cells was enhanced after upregulation of miR-21a-5p (Fig. 5I-J, M) but weakened in the miR-21a-5p knockdown group (Fig. 5K-L, M). In addition, miR-21a-5p overexpression slightly increased A1s markers without CNTF pre-treatment, while downregulating miR-21a-5p slightly decreased A1s markers and increased A2s markers. These data illustrated that miR-21a-5p could repress the inhibitory effect of CNTF on A1s induction.

**Figure 6:**
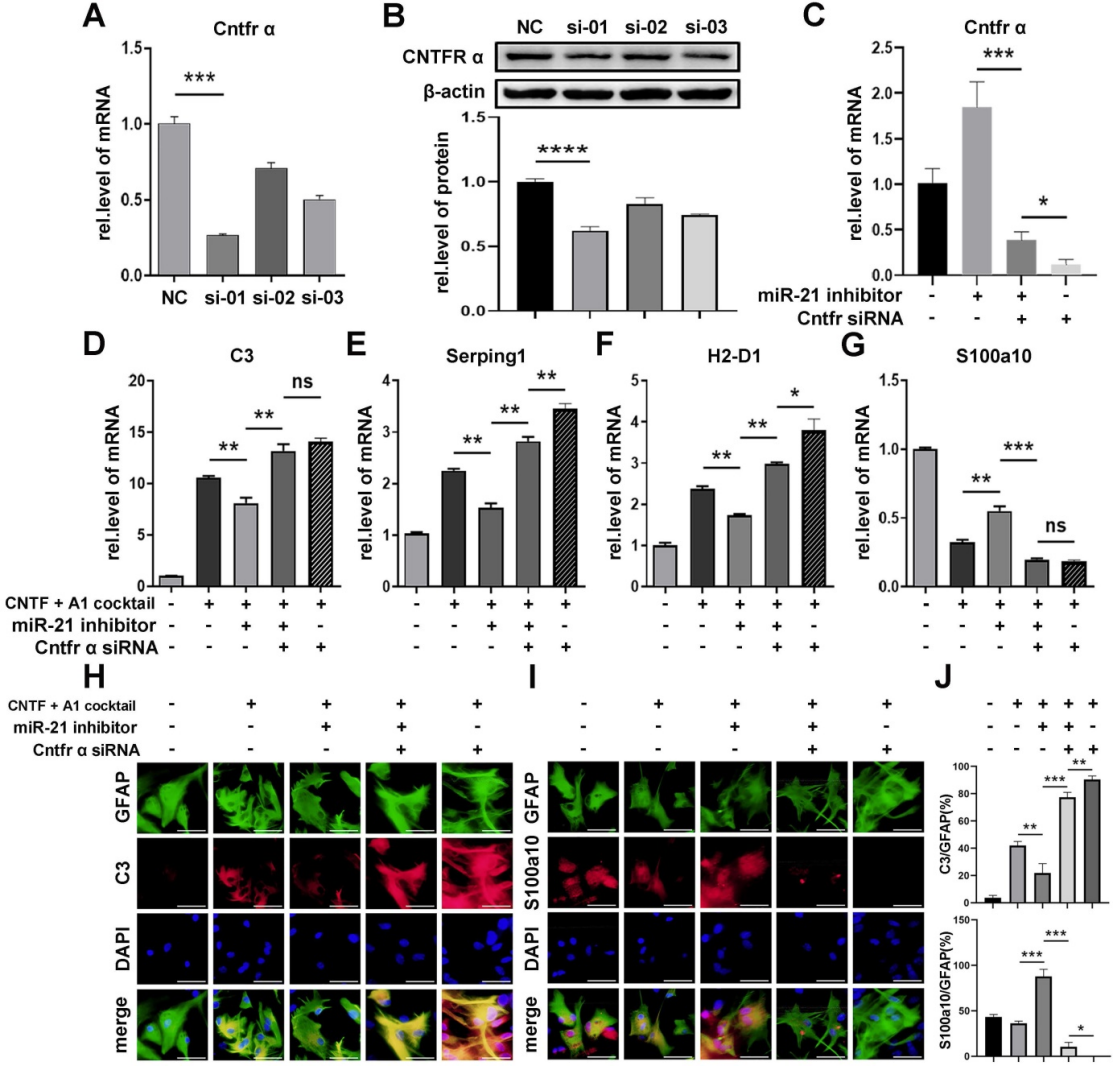
** miR-21a-5p inhibits the effect of CNTF by inhibiting the expression of *Cntfr α*.** Three types of Cntfr α siRNA were transfected into astrocytes. The expression of *Cntfr α* mRNA was detected by qRT-PCR (**A**). The expression of CNTFR α protein was detected by western blotting, and the results were analyzed with Image J, GraphPad, and SPSS (**B**). (**C**) miR-21 inhibitor, *Cntfr α* siRNA, and negative controls were transfected into astrocytes, and the expression of *Cntfr α* mRNA was detected by qRT-PCR, normalized to GAPDH. (**D-G**) miR-21 inhibitor, *Cntfr α* siRNA, and negative controls were transfected into astrocytes. After pre-treating with CNTF, astrocytes were induced into A1s. The expression of *C3, Serping1, H2D1,* and* S100a10* mRNA were detected by qRT-PCR and normalized to GAPDH. The results were analyzed with GraphPad and SPSS. The data are expressed as mean ±SD, n=3. *p <0.05, **p <0.01, ***p <0.001. (**H-I**) Immunofluorescence was used to detect GFAP (green; **H-**I), C3 (red; **H**), S100a10 (red; **I**) and DAPI (blue; nuclei), n=3; the results were analyzed with Image J, GraphPad, and SPSS (**J**). *p <0.05, **p <0.01, ***p <0.001.

To determine whether miR-21a-5p inhibited the function of CNTF by targeting *Cntfr α*, we transfected *Cntfr α* siRNA into A1s together with miR-21a-5p knockdown. First, we chose the siRNA containing the highest transfection efficiency by qRT-PCR and western blotting assays (Fig. 6A-B). As shown in Fig. 6C, cells transfected with *Cntfr α* siRNA showed a lower level of *Cntfr α* compared to those co-transfected with a miR-21a-5p inhibitor. Significantly, qRT-PCR revealed that the miR-21a-5p knockdown enhanced the inhibitory effect of CNTF on A1s induction, which was reversed by *Cntfr α* downregulation (Fig. 6D-G). Immunofluorescence staining further confirmed that co-transfection with miR-21a-5p inhibitor and *Cntfr α* siRNA exhibited increased C3 expression and decreased S100a10 expression in GFAP^+^ cells compared with cells treated with miR-21a-5p inhibitor (Fig. 6H-J). We also detected the level of A1/A2 markers after cells were transfected with *Cntfr α* siRNA to elucidate the function of Cntfr α, whether it was affected by miR-21a-5p or not. Importantly, qRT-PCR and immunofluorescence staining showed higher A1 and lower A2 marker expression compared with the downregulated miR-21a-5p group.

In general, miR-21a-5p suppressed the function of CNTF on A1s induction by downregulating *Cntfr α*.

### miR-21 promoted A1s induction by down-regulating Cntfr α *in vivo*

**Figure 7:**
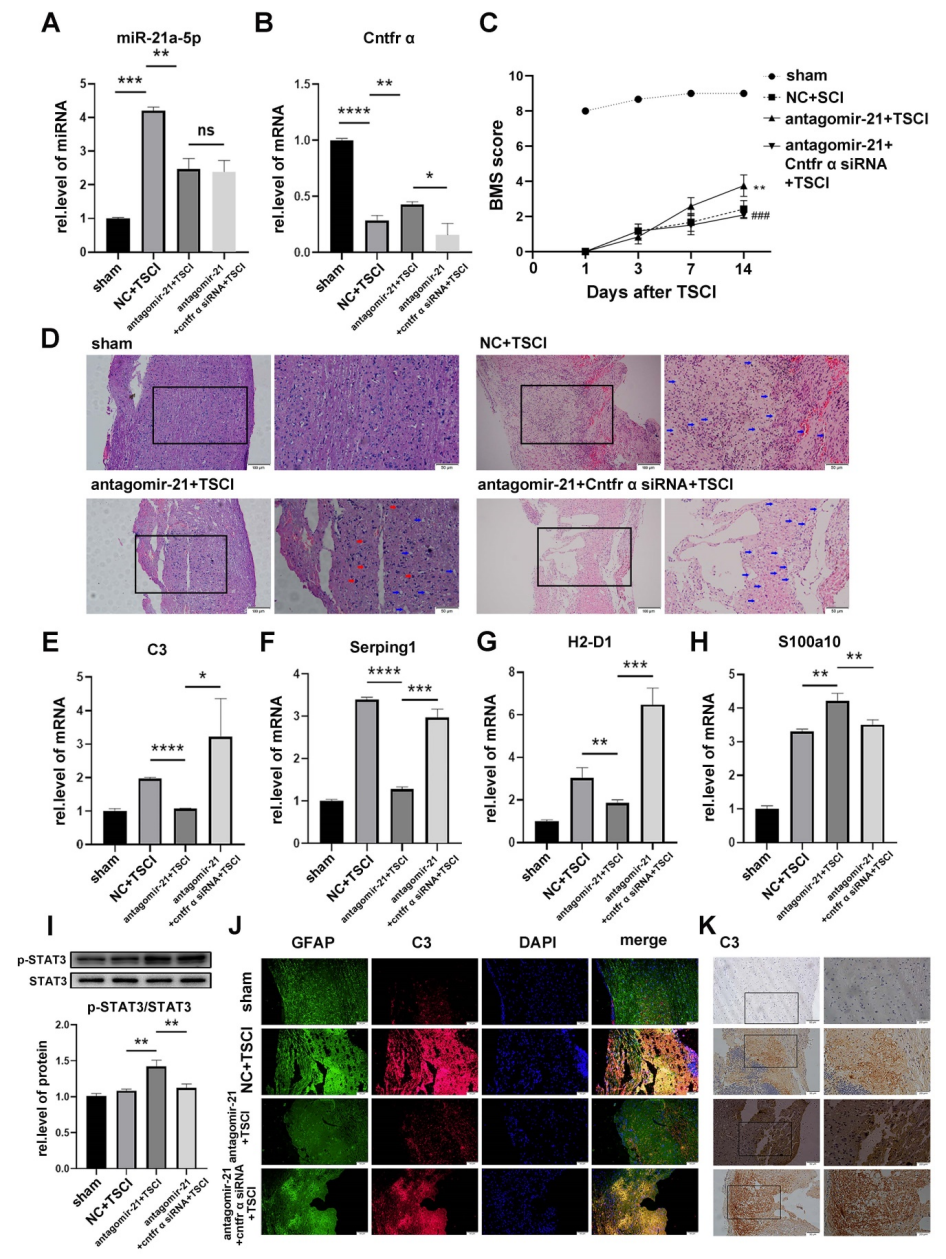
** miR-21 promotes the polarization of neurotoxic astrocytes (A1) by inhibiting the expression of Cntfr α *in vivo***. A total of 96 mice were divided into 4 groups: sham (n=24), NC+TSCI (n=24), antagomir-21+TSCI (n=24), antagomir-21+*Cntfr α* siRNA+TSCI (n=24). (**A**) miR-21 expression was detected by qRT-PCR and normalized by U6. (**B**) The expressions of *Cntfr α* was detected by qRT-PCR and normalized to GAPDH. Results were analyzed by GraphPad and SPSS. Data are expressed as mean ±SD, n=6. *p <0.05, **p <0.01, ***p <0.001, ****p <0.0001. (**C**) BMS scores indicated the motor function over 14 days after TSCI. Results are shown as mean ±SD (NC+SCI VS antagomir-21+SCI: **p <0.001; antagomir-21+SCI VS antagomir-21+Cntfr α siRNA+SCI: ^###^p <0.001). (**D**) Hematoxylin and eosin staining (H&E staining) of the spinal cord tissue in the sham group and SCI groups at 14d post-surgery. (**E-H**) The expression of *C3, Serping1, H2D1,* and* S100a10* mRNA were detected by qRT-PCR and normalized to GAPDH. Results were analyzed with GraphPad and SPSS. Data are expressed as mean ±SD, n=6. *p <0.05, **p <0.01, ***p <0.001, ****p <0.0001. (**J**) Western blotting was used to detect the expression of p-STAT3, STAT3, and β-actin protein. The results were analyzed with Image J, GraphPad, and SPSS software. Data are expressed as mean ±SD, n=6. *p <0.05, **p <0.01, ***p <0.001, ****p <0.0001. (**K**) Immunofluorescence was used to detect GFAP (green), C3 (red), and DAPI (blue; nuclei). Scale bar, 50 µm. n=6 (**L**) Immunohistochemistry was used to detect C3. Scale bar, low magnification: 50 µm; high magnification: 20 µm. n=6.

To confirm whether miR-21a-5p regulated the polarization of reactive astrocytes *in vivo*, antagomir-21 was used to regulate the expression of miR-21a-5p in *Cntfr α* siRNA-treated mice. As shown in Fig. 7A-B, the expression of miR-21a-5p in the antagomir-21 and antagomir-21+ *Cntfr α* siRNA group was decreased, while the expression of Cntfr α was increased in the antagomir-21 group; this effect was abolished by Cntfr α knockdown. To demonstrate the effect of miR-21a-5p/Cntfr α in TSCI, BMS scores were used for assessing the hindlimb motor function from 1 d post-TSCI. In the sham group, the motor function showed a recovery after 2 d, and returned to normal after 4 d post-surgery. The antagomir-21+TSCI group showed better recovery 7 d after TSCI, and more significant improvement in motor function was observed 14 d post-TSCI compared with the NC+TSCI group. Significantly, the antagomir-21+*Cntfr α* siRNA+TSCI group showed poor functional recovery (Fig. 7C). As shown in Fig. 7D, H&E staining revealed the damaged structural integrity on 14 d post-surgery spinal cord in the NC+TSCI group, which included increased vacuoles (indicated by blue arrows) and neural degeneration. In the antagomir-21+TSCI group, results showed fewer vacuoles (indicated by blue arrows) and neural regeneration (indicated by red arrows) to some extent while the antagomir-21+*Cntfr α* siRNA+TSCI group showed the opposite result. These data indicated that miR-21a-5p/Cntfr α has a significant functional regulating effect after TSCI. A qRT-PCR assay on 3 d post-TSCI showed decreased *C3, Serping1*, and *H2-D1* expression and increased *S100a10* after treatment with antagomir-21, which can be reversed by *Cntfr α* siRNA treatment (Fig. 7E-H), indicating that miR-21a-5p could promote A1s induction by decreasing Cntfr α *in vivo*. In the early stage of TSCI, the stimulation caused by ischemic injury might affect the polarization of reactive astrocytes in addition to neuroinflammation, as there was higher S100a10 expression in the NC TSCI group than in the sham group (Fig. 7H). Subsequently, western blotting showed that the phosphorylation of STAT3 was significantly increased by antagomir-21 treatment but decreased after treatment with *Cntfr α* siRNA after TSCI (Fig. 7I), which demonstrated that miR-21a-5p could affect the STAT3 signaling pathway by downregulating *Cntfr α* after TSCI.

Furthermore, immunofluorescence staining on 3 d post-TSCI showed that C3 expression in GFAP^+^ cells was significantly downregulated by antagomir-21 treatment, and was recused by Cntfr α knockdown (Fig. 7J). The same trend was observed in the immunohistochemistry assay (Fig. 7K).

These data confirmed that miR-21a-5p promotes the induction of A1s by downregulating Cntfr α after TSCI *in vivo*.

## Discussion

To date, there has been no effective treatment for axon degeneration, which is the main reason underlying the poor prognosis of TSCI 1. Promoting axonal regeneration and anti-inflammation are often used to treat TSCI in the clinic 3.

Reactive astrogliosis is a key part of the pathological process of traumatic spinal cord injury and is of great significance to the progress of its treatment. Previous studies have found that reactive astrocytes exhibit different (harmful or beneficial) phenotypes under different stimuli (inflammation or ischemia). This shows that, besides the commonness of proliferation, reactive astrogliosis has a high heterogeneity in specific injuries 16. Recently, Liddelow et al. reported that there were two subtypes of reactive astrocytes, termed A1s and A2s 18. A1s, neurotoxic reactive astrocytes, are induced by reactive microglia (lipopolysaccharide-stimulated) that release IL-1α, TNF-α, and C1q. A1s lose their ability for neuroprotection, synaptogenesis, and phagocytosis, thereby inducing apoptosis of neurons and oligodendrocytes. Blocking A1s induction prevents the death of axotomized neurons 18. In contrast, A2s, which are induced by ischemia, could strongly exert a neuroprotective effect. Thus, it is necessary to regulate the alteration of reactive astrocytes from A1s to A2s, and then suppress inflammation for TSCI recovery.

**Figure 8:**
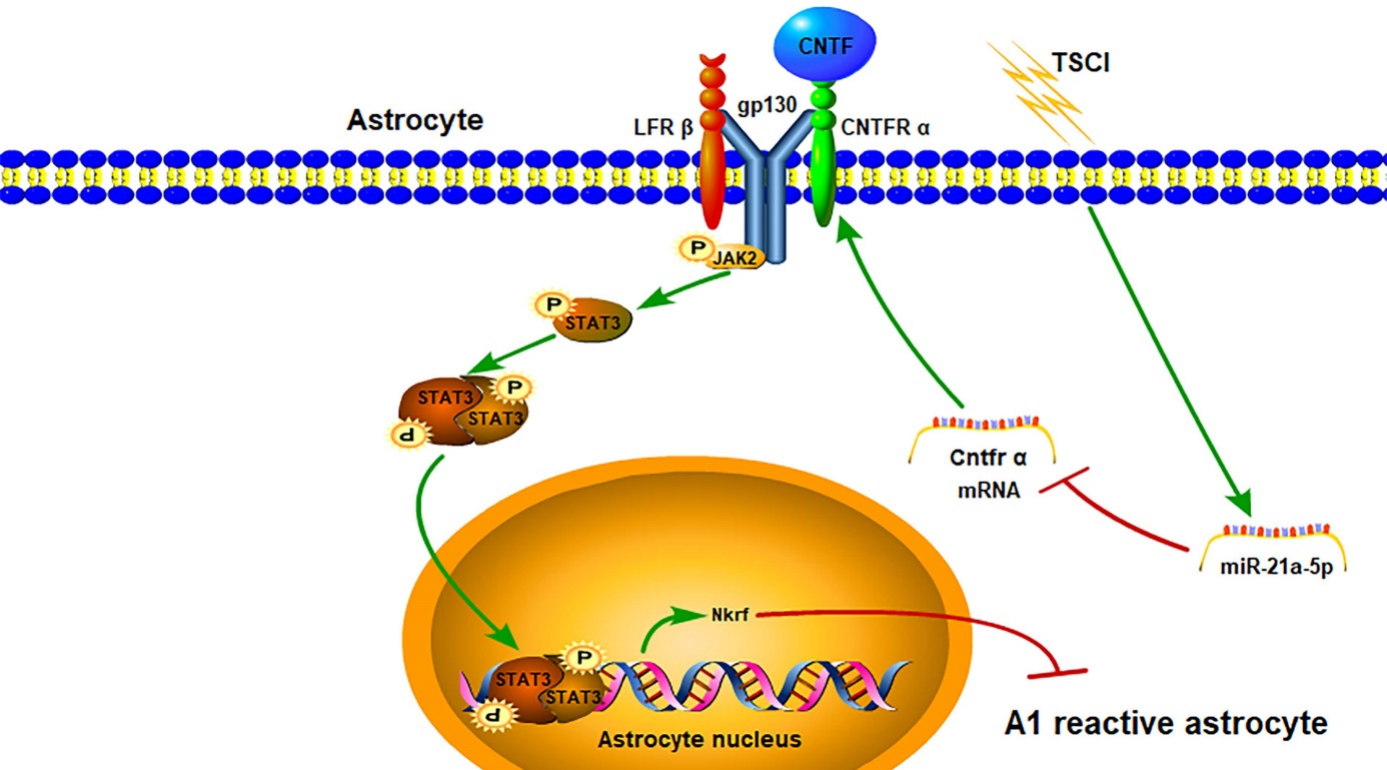
The miR-21a-5p pathway regulates the polarization of reactive astrocytes via the Cntfr α/STAT3/Nkrf axis after TSCI.

In our study, we confirmed for the first time that miR-21a-5p could promote the induction of A1 reactive astrocytes via the CNTF/STAT3/Nkrf pathway after traumatic spinal cord injury (Fig. 8). Our study confirmed that A1s induction could be simulated by the TSCI model *in vivo*. Astrocytes were also transformed into A1s by treating with IL-1α, TNF-α, and C1q *in vitro*. Based on this, the aim of our study was to explore key molecules that regulate A1s polarization.

Our previous studies described that miR-21a-5p regulated glial scar formation 45 and inhibited the polarization of astrocytes into A2s in ISCI 20. However, the effect of miR-21a-5p on neurotoxic reactive astrocytes (A1s) in TSCI has not been clarified. Interestingly, we found that miR-21a-5p was upregulated in A1s both *in vivo* and *in vitro*, suggesting it may be an important factor for the polarization of reactive astrocytes in TSCI.

Concerning the potential mechanism of the miR-21a-5p-related reactive astrocyte polarization, a gene chip assay and bioinformatics analysis were used to explore target genes that could mediate the polarization of reactive astrocytes. Subsequently, we found that four genes may contain a binding site for miR-21a-5p. Upon further analysis, *Cntfr α* was confirmed to be targeted by miR-21a-5p. Cntfr α is a specific receptor of ciliary neurotrophic factor (CNTF), which is expressed in neurons, microglia, and astrocytes. Furthermore, it activates the classical STAT3 signal pathway by binding with CNTF. Interestingly, when astrocytes were treated with CNTF after upregulation of miR-21a-5p, the STAT3 signal pathway was weakened. Therefore, miR-21a-5p could downregulate the CNTF/STAT3 pathway and might be essential for regulating the induction of A1s. Nevertheless, it remains unclear how CNTF/STAT3 regulates the polarization of reactive astrocytes.

Previous studies confirmed the positive effect of CNTF on reactive astrogliosis 25, 30-32 and M2 macrophage induction through activation of the classical STAT3 signal pathway 35. Considering the important regulatory role of STAT3, we speculated that CNTF could regulate the polarization of reactive astrocytes from A1s to A2s and might promote A2-type reactive astrogliosis by activating the STAT3 signaling pathway. In our study, we demonstrated that CNTF could downregulate A1 markers and upregulate A2 markers *in vitro*, which would be inhibited by repressing the activation of STAT3. However, it remains unclear how the transcription factor STAT3 affects A1 induction. The NF-κB signaling pathway is important for the polarization of A1 reactive astrocytes 17, 36, 37. Moreover, NF-κB repressing factor (Nkrf), a specific inhibitor of the NF-κB signaling pathway 49-51, might be related to the induction of M1 microglia and release of microglia-induced neuroinflammatory factors 52. However, the effect of Nkrf in reactive astrocytes remains unclear. Significantly, we found that CNTF could regulate the expression of *Nkrf* by promoting the transcription of STAT3, which was confirmed by qRT-PCR and ChIP assays. These data reveal a novel mechanism by which CNTF regulates A1s polarization through the STAT3/Nkrf pathway.

As miR-21a-5p is a key molecule functioning upstream molecule of *Cntfr α*, it was of great significance to verify whether miR-21a-5p could affect the function of CNTF/CNTFR α. Our data showed that the effect of CNTF on A1s was strongly enhanced after down-regulating the expression of miR-21a-5p. Importantly, these effects were reduced when Cntfr α was knocked down. Downregulating miR-21a-5p could also markedly regulate the polarization of reactive astrocytes, which was reversed by Cntfr α knockdown in a mouse TSCI model. Moreover, BMS score and H&E staining demonstrated miR-21a-5p/Cntfr α could control neural regeneration and the locomotor functional recovery through regulation of neurotoxic reactive astrocytes (A1s) after TSCI in mice. Interestingly, we also found that A1/A2 markers were slightly affected by miR-21a-5p even without CNTF treatment in A1s. Further studies will be performed to determine the underlying mechanism. Thus, we propose miR-21a-5p may be a key factor for targeted treatment of TSCI in the future.

Our study indicated that the miR-21a-5p/Cntfr α axis could regulate reactive astrocytes polarization through STAT3 signaling. In a previous study, Herrmann et al. reported that conditional knockout of the STAT3 in astrocytes could inhibit astrocyte hypertrophy, disrupt astroglial scar formation, and then restrain neural regeneration after spinal cord injury 32. This significant study confirmed that STAT3 signaling is a critical factor for reactive astrogliosis and scar formation, which offers profound guidance for future research on reactive astrocytes. Recently, reactive astrocytes have been divided into multiple subtypes but mainly neurotoxic or neuroprotective reactive astrocytes (A1s or A2s). Based on these, we speculated that A2s might be the main subtype of astroglial scars, in which exert the neuroprotective effect in CNS disease. Blocking STAT3 signaling might inhibit neuroprotective reactive astrogliosis, and transform them into the neurotoxic subtype. In our study, inhibition of the miR-21a-5p/Cntfr α axis could decrease neurotoxic reactive astrocytes, and increase neuroprotective astroglial scar formation. In the future, we will focus on multiple subtypes of reactive astrocytes to further explore the significance.

Taken together, our results confirmed that astrocyte-mediated neuroinflammation was regulated by miR-21a-5p. This is an innovative point of our research showing the effect of miR-21a-5p on astrocytic neuroinflammation after TSCI from another perspective. In addition, regulating miR-21a-5p may improve the environment for neuro-regeneration.

However, our study only examined the expression of A1/A2 marker genes, but did not detect other astrocytic functions, such as activity, migration, and effects on neurons. In addition, we demonstrated that CNTF inhibited A1s polarization by directly activating Nkrf. These factors underlying the alteration of reactive astrocytes to A2 by miR-21a-5p/CNTF/STAT3 will be the focus of future studies.

Many studies showed that it is necessary for the recovery of TSCI to promote the alteration of reactive astrocytes from A1s to A2s. However, it remains to be determined whether other factors affect the alteration of A1/A2 reactive astrocytes. Our study only analyzed the transcript in the overall spinal cord, and a specific analysis of the astrocyte population needs to be performed. These issues can be addressed through single-cell sequencing, proteomics, or other methods.

Collectively, our study confirmed that miR-21a-5p could promote the induction of A1s through the Cntfr α/STAT3/Nkrf axis after TSCI; this finding may provide insight into reactive astrocyte alteration and promote the development of TSCI recovery techniques.

## Supplementary Material

Supplementary figures and tables.

## Figures and Tables

**Table 1:** Sequence of primer pairs used in the study

Gene	Primer sequence, 5'-3'	
	Forward	Reverse
mmu-miR-21a-5p	CCTAGCTTATCAGACTGATGTTGA	
U6	GGAACGATACAGAGAAGATTAGC	TGGAACGCTTCACGAATTTGCG
Cntfr α	TCCCAGGAAGACTTTGGTCTGG	CTGTGGACTGTGTTTCTGCGTGT
C3	GCAGACCTTAGCGACCAAGT	CCGCAATGACTGTTGGTGTC
Serping1	TACGATCTCAACCTGTGCGG	AGTTCCAGCACTGTCTCGTG
H2-D1	GCATTACAAGGCCTACCTGGA	CAGCACCTCAGGGTGACTTC
S100a10	TACGTTTCACAGGTTTGCAGG	TCCCGTTCCATGAGCACTCT
iNOS	CCTGCTTTGTGCGAAGTGTC	CCCAAACACCAAGCTCATGC
Il-1β	TCCAGGATGAGGACATGAGCAC	GAACGTCACACACCAGCAGGTTA
Nkrf	CACTCAGGCTCTTCACCCAA	GCGGAGACCTGTCATCCTTT
Epha4	GCAATCCCAACAGCCTGAAGA	CAGCCAGTCGCCCACTGATA
Pitx2	AACCTTACGGAAGCCCGAGTC	CCCAAAGCCATTCTTGCACA
Abad2	TGACCAAGACCTAGAACGCATCC	TTTCCAGTCCATGACTGCATCC
GAPDH	TGTCTCCTGCGACTTCAACA	GGTGGTCCAGGGTTTCTTACT

## Abbreviations

TSCI: Traumatic Spinal Cord Injury
miR: microRNA
CNTF: ciliary neurotrophic factor
CNTFR α: ciliary neurotrophic factor receptor α
CNS: central nervous system
A1s: neurotoxic reactive astrocyte
A2s: neurotrophic reactive astrocyte
NC: negative control
siRNA: small interfering RNA
PBS: phosphate-buffered saline
IL-1α: interleukin 1α
TNF-α: tumor necrosis factor - α
C1q: complement component 1q
STAT3: signal transducer and activator of transcription-3
GFAP: glial fibrillary acidic protein
C3: complement component 3
Serping1: serpin family G member 1
H2-D1: histocompatibility 2, D region locus 1
S100a10: S100 calcium-binding protein A10
IL-1β: interleukin 1β
iNOS: inducible nitric oxide synthase
GAPDH: glyceraldehyde 3-phosphate dehydrogenase
DAPI: 4',6-diamidino-2-phenylindole
NF-κB: nuclear factor-κB
Nkrf: NF-κB repressing factor
qRT-PCR: quantitative real-time polymerase chain reaction
ELISA: Enzyme-linked immunosorbent assay
ChIP: chromatin immunoprecipitation
BMS: Basso Mouse Scale
