# Supplementary material for: miR-21a-5p Promotes Inflammation following Traumatic Spinal Cord Injury through Upregulation of Neurotoxic Reactive Astrocyte (A1) Polarization by Inhibiting the CNTF/STAT3/Nkrf Pathway
Source: Int J Biol Sci. 2021 Jul 5;17(11):2795–810. doi: 10.7150/ijbs.60509 (PMC8326122; doi:10.7150/ijbs.60509)
Supplement: Supplementary file 1 — Supplementary figures and tables. [file ijbsv17p2795s1.zip › Supplementary materials/Supplementary materials.docx]

**Supplementary information**

**Table. S1.** siRNA sequence used in the study.

| Gene | Sequence of siRNA |
| --- | --- |
| si-m-Cntfr_001 | CCAACAATCTCCTGATCTG |
| si-m-Cntfr_002 | CACCTGTTCTCAACCATCA |
| si-m-Cntfr_003 | AATCCTTTCCTCTCAAGTT |

**Table. S2.** Sequence of Vector for RNA pulldown

|  | Vector for RNA pulldown; Vector: pcDNA3.1(+) |
| --- | --- |
| mus-cntfr in pcDNA3.1(+) | GAGCTCCCTCGGGCTGGACCCTCCAACGCCAGCGACTCCCAGGAGCCCTTGGGGGACCTGAGGGGAGCCCCCACCCCACATCCACAGTTTTCTCCTCCTGCCCCAGCCTCCTGTCTGTCCCAGGGTCTTTGTTGCCACCATCAGATTATAAGCTCCTGACACTGGGGGGGCCCAGCCATCCCCCTCCCCCCGGTGCCCACACTTTTCAGTCCTTCCACCTTTGCCCCTGTTTTGTACGATCCTCCATTGGCCCTTTCCTACCCCCCAGTATTTAATGTCCTGTCAGTCCCTTCTAGTCTGACTCAATGGTAACGTCGAC |
| antisense-MUT-  cntfr in pcDNA3.1(+) | CAGCTGCAATGGTAACTCAGTCTGATCTTCCCTGACTGTCCTGTAATTTATGACCCCCCATCCTTTCCCGGTTACCTCCTAGCATGTTTTGTCCCCGTTTCCACCTTCCTGACTTTTCACACCCGTGGCCCCCCTCCCCCTACCGACCCGGGGGGGTCACAGTCGAAATTGCTTAGACTACCACCGTTGTTTCTGGGACCCTGTCTGTCCTCCGACCCCGTCCTCCTCTTTTGACACCTACACCCCACCCCCGAGGGGAGTCCAGGGGGTTCCCGAGGACCCTCAGCGACCGCAACCTCCCAGGTCGGGCTCCCTCGAG |

**Table. S3.** Sequence of Vector for Dual-luciferase reporter

|  | Vector for Dual-luciferase reporter; Vector: pmirGLO |
| --- | --- |
| PmirGLO-Cntfr-3’UTR（WT） | GAGCTCCCTCGGGCTGGACCCTCCAACGCCAGCGACTCCCAGGAGCCCTTGGGGGACCTGAGGGGAGCCCCCACCCCACATCCACAGTTTTCTCCTCCTGCCCCAGCCTCCTGTCTGTCCCAGGGTCTTTGTTGCCACCATCAGATTATAAGCTCCTGACACTGGGGGGGCCCAGCCATCCCCCTCCCCCCGGTGCCCACACTTTTCAGTCCTTCCACCTTTGCCCCTGTTTTGTACGATCCTCCATTGGCCCTTTCCTACCCCCCAGTATTTAATGTCCTGTCAGTCCCTTCTAGTCTGACTCAATGGTAACGTCGAC |
| PmirGLO-Cntfr-3’UTR（MUT） | GAGCTCCCTCGGGCTGGACCCTCCAACGCCAGCGACTCCCAGGAGCCCTTGGGGGACCTGAGGGGAGCCCCCACCCCACATCCACAGTTTTCTCCTCCTGCCCCAGCCTCCTGTCTGTCCCAGGGTCTTTGTTGCCACCATCAGATTCGTTAAAGCTGACACTGGGGGGGCCCAGCCATCCCCCTCCCCCCGGTGCCCACACTTTTCAGTCCTTCCACCTTTGCCCCTGTTTTGTACGATCCTCCATTGGCCCTTTCCTACCCCCCAGTATTTAATGTCCTGTCAGTCCCTTCTAGTCTGACTCAATGGTAACGTCGAC |

**
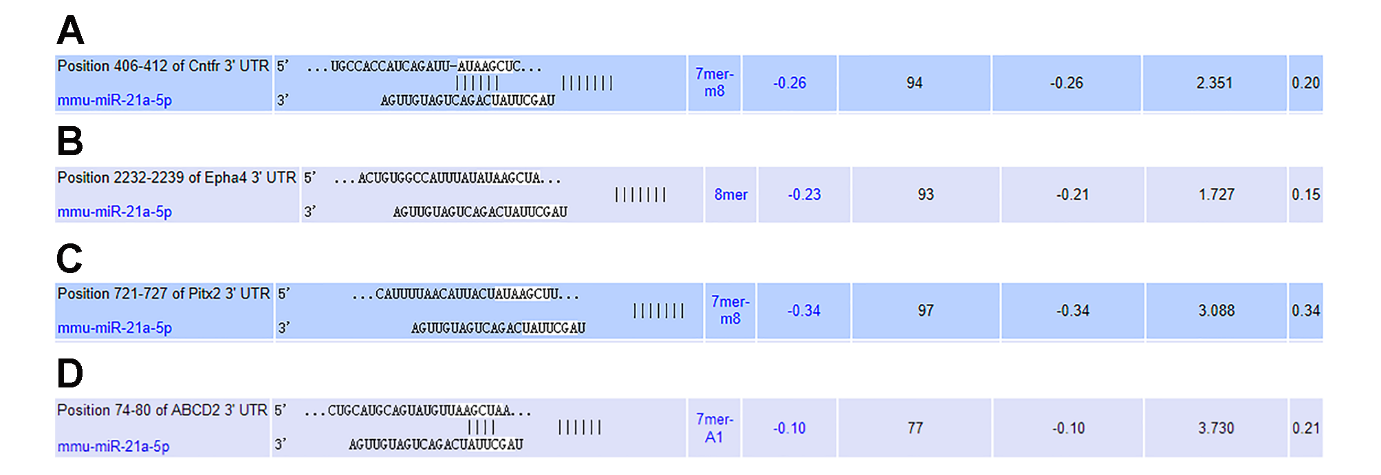
**

**Fig. S1.** Prediction of putative binding sites between miR-21a-5p and Cntfr α (A), Epha4 (B), Pitx2 (C), Abcd2 (D).

**
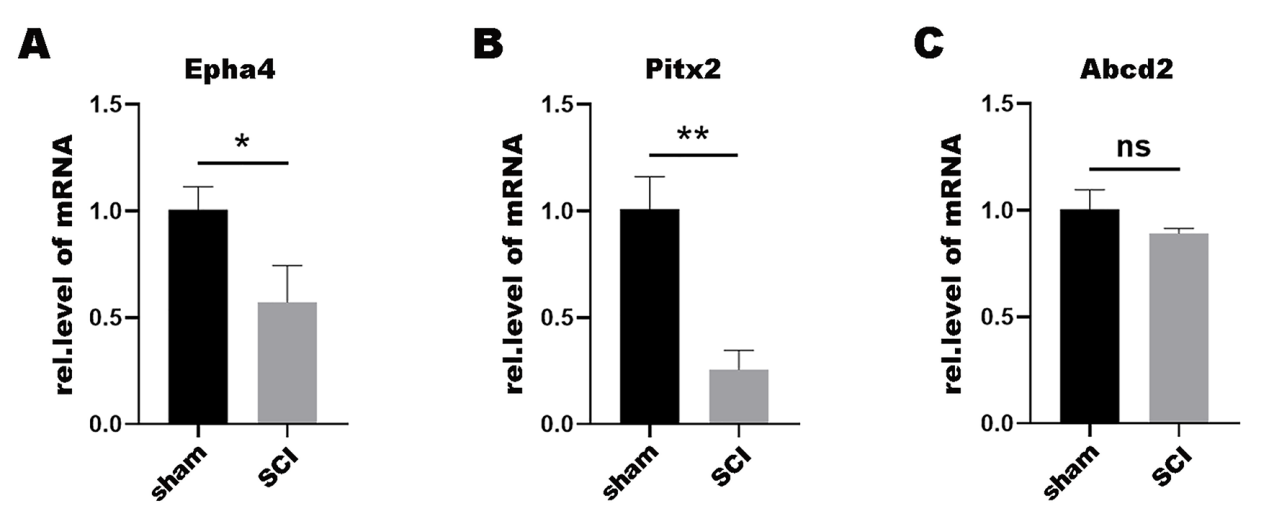
**

**Fig. S2.** Expression of mRNAs after TSCI. (A-C). qRT-PCR was used to detect the expression of Epha4, Pitx2, and Abcd2 between the sham group and 3d post-TSCI group, and GAPDH was used to normalize it.

**
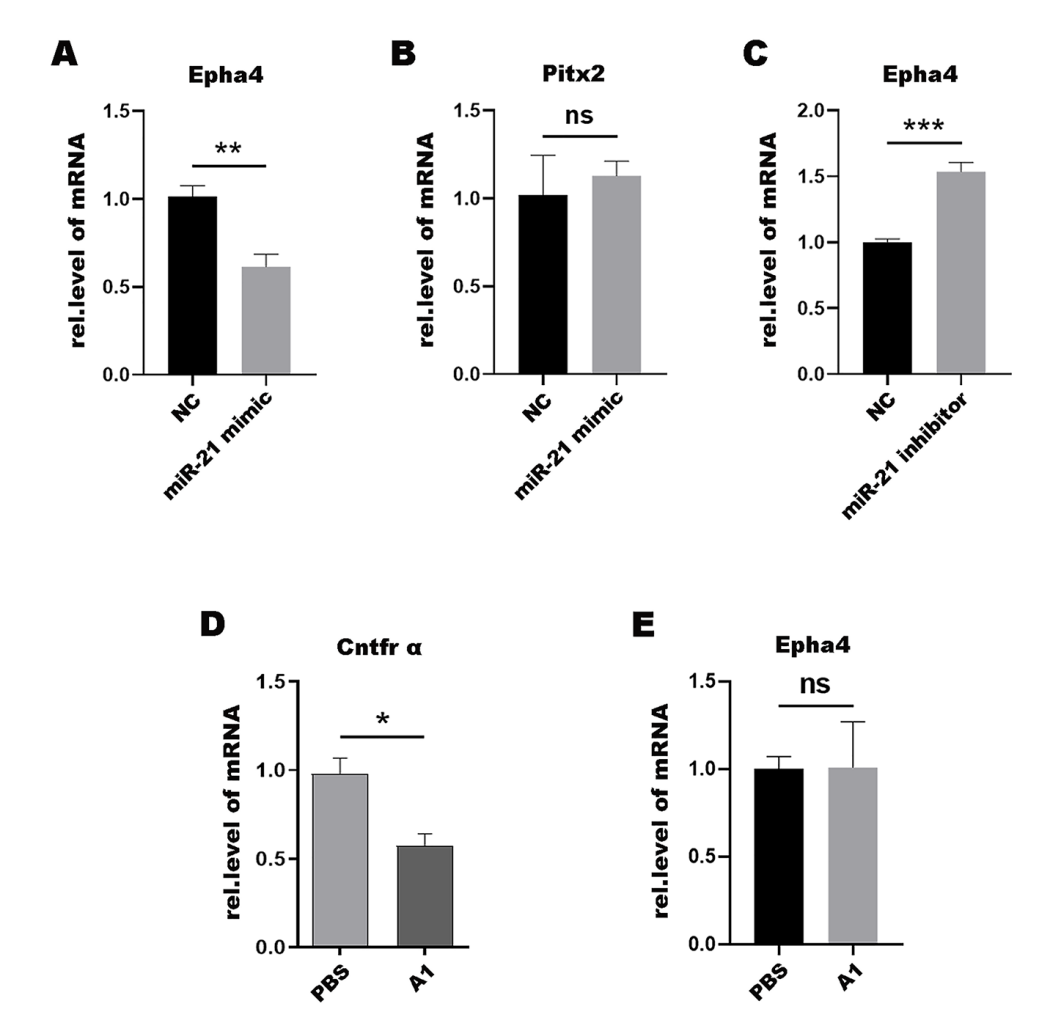
**

**Fig. S3.** Effect of miR-21a-5p on mRNA expression in astrocytes. The miR-21a-5p mimic and negative control were transfected into astrocytes. (A, B). qRT-PCR was used to detect the mRNA levels of Epha4 and Pitx2, normalized to GAPDH. (C). The miR-21a-5p inhibitor and negative control were transfected into astrocytes. qRT-PCR was used to detect the mRNA level of Epha4, normalized to GAPDH. (D, E) Astrocytes were induced to A1s. qRT-PCR was used to detect the expression of Cntfr α and Epha4, normalized to GAPDH.
