# Supplementary material for: miR-21a-5p Promotes Inflammation following Traumatic Spinal Cord Injury through Upregulation of Neurotoxic Reactive Astrocyte (A1) Polarization by Inhibiting the CNTF/STAT3/Nkrf Pathway
Source: Int J Biol Sci. 2021 Jul 5;17(11):2795–810. doi: 10.7150/ijbs.60509 (PMC8326122; doi:10.7150/ijbs.60509)
Supplement: Supplementary file 1 — Supplementary figures and tables. [file ijbsv17p2795s1.zip › Supplementary materials/Supplementary Table.docx]

**Table. S1.** Sequence of siRNA used in the study.

| Gene | Sequence of siRNA |
| --- | --- |
| si-m-Cntfr_001 | CCAACAATCTCCTGATCTG |
| si-m-Cntfr_002 | CACCTGTTCTCAACCATCA |
| si-m-Cntfr_003 | AATCCTTTCCTCTCAAGTT |

**Table. S2.** Sequence of Vector for RNA pulldown

|  | Vector for RNA pulldown; Vector: pcDNA3.1(+) |
| --- | --- |
| mus-cntfr in pcDNA3.1(+) | GAGCTCCCTCGGGCTGGACCCTCCAACGCCAGCGACTCCCAGGAGCCCTTGGGGGACCTGAGGGGAGCCCCCACCCCACATCCACAGTTTTCTCCTCCTGCCCCAGCCTCCTGTCTGTCCCAGGGTCTTTGTTGCCACCATCAGATTATAAGCTCCTGACACTGGGGGGGCCCAGCCATCCCCCTCCCCCCGGTGCCCACACTTTTCAGTCCTTCCACCTTTGCCCCTGTTTTGTACGATCCTCCATTGGCCCTTTCCTACCCCCCAGTATTTAATGTCCTGTCAGTCCCTTCTAGTCTGACTCAATGGTAACGTCGAC |
| antisense-MUT-  cntfr in pcDNA3.1(+) | CAGCTGCAATGGTAACTCAGTCTGATCTTCCCTGACTGTCCTGTAATTTATGACCCCCCATCCTTTCCCGGTTACCTCCTAGCATGTTTTGTCCCCGTTTCCACCTTCCTGACTTTTCACACCCGTGGCCCCCCTCCCCCTACCGACCCGGGGGGGTCACAGTCGAAATTGCTTAGACTACCACCGTTGTTTCTGGGACCCTGTCTGTCCTCCGACCCCGTCCTCCTCTTTTGACACCTACACCCCACCCCCGAGGGGAGTCCAGGGGGTTCCCGAGGACCCTCAGCGACCGCAACCTCCCAGGTCGGGCTCCCTCGAG |

**Table. S3.** Sequence of Vector for Dual-luciferase reporter

|  | Vector for Dual-luciferase reporter; Vector: pmirGLO |
| --- | --- |
| PmirGLO-Cntfr-3’UTR（WT） | GAGCTCCCTCGGGCTGGACCCTCCAACGCCAGCGACTCCCAGGAGCCCTTGGGGGACCTGAGGGGAGCCCCCACCCCACATCCACAGTTTTCTCCTCCTGCCCCAGCCTCCTGTCTGTCCCAGGGTCTTTGTTGCCACCATCAGATTATAAGCTCCTGACACTGGGGGGGCCCAGCCATCCCCCTCCCCCCGGTGCCCACACTTTTCAGTCCTTCCACCTTTGCCCCTGTTTTGTACGATCCTCCATTGGCCCTTTCCTACCCCCCAGTATTTAATGTCCTGTCAGTCCCTTCTAGTCTGACTCAATGGTAACGTCGAC |
| PmirGLO-Cntfr-3’UTR（MUT） | GAGCTCCCTCGGGCTGGACCCTCCAACGCCAGCGACTCCCAGGAGCCCTTGGGGGACCTGAGGGGAGCCCCCACCCCACATCCACAGTTTTCTCCTCCTGCCCCAGCCTCCTGTCTGTCCCAGGGTCTTTGTTGCCACCATCAGATTCGTTAAAGCTGACACTGGGGGGGCCCAGCCATCCCCCTCCCCCCGGTGCCCACACTTTTCAGTCCTTCCACCTTTGCCCCTGTTTTGTACGATCCTCCATTGGCCCTTTCCTACCCCCCAGTATTTAATGTCCTGTCAGTCCCTTCTAGTCTGACTCAATGGTAACGTCGAC |
