# Supplementary figures and images for: miR-21a-5p Promotes Inflammation following Traumatic Spinal Cord Injury through Upregulation of Neurotoxic Reactive Astrocyte (A1) Polarization by Inhibiting the CNTF/STAT3/Nkrf Pathway
Source: Int J Biol Sci. 2021 Jul 5;17(11):2795–810. doi: 10.7150/ijbs.60509 (PMC8326122; doi:10.7150/ijbs.60509)

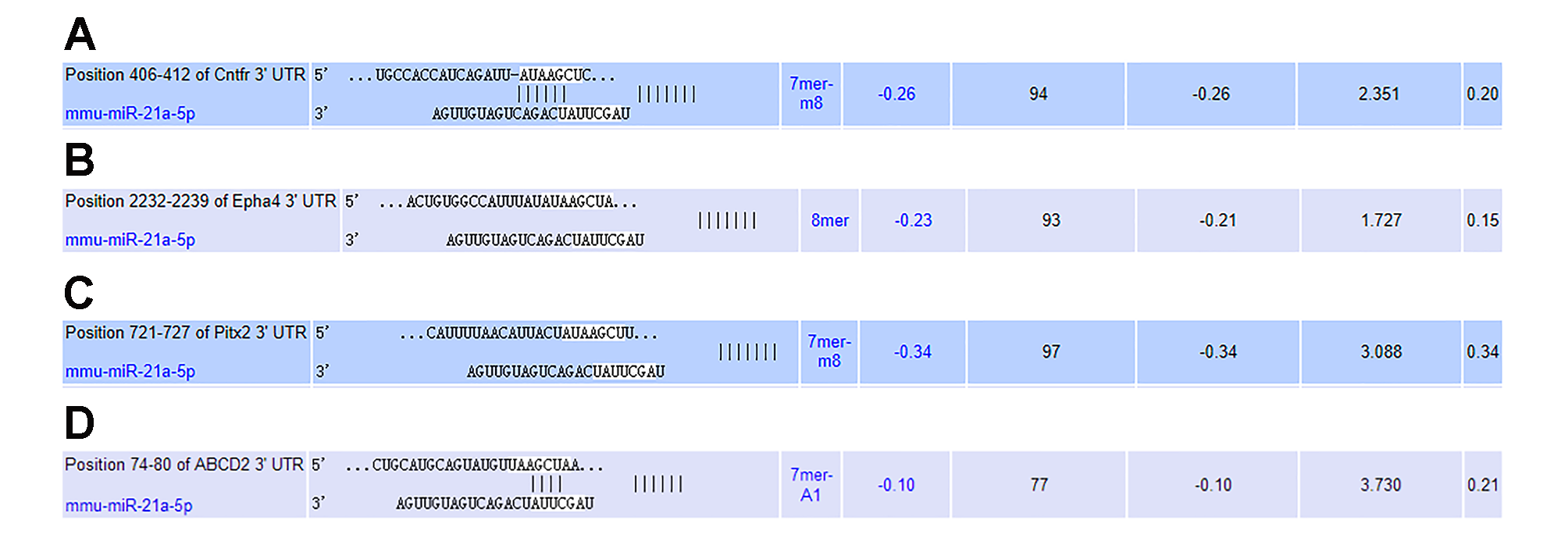

Supplement: Supplementary file 1 — Supplementary figures and tables. [file ijbsv17p2795s1.zip › Supplementary materials/zyn sfig1.tif]

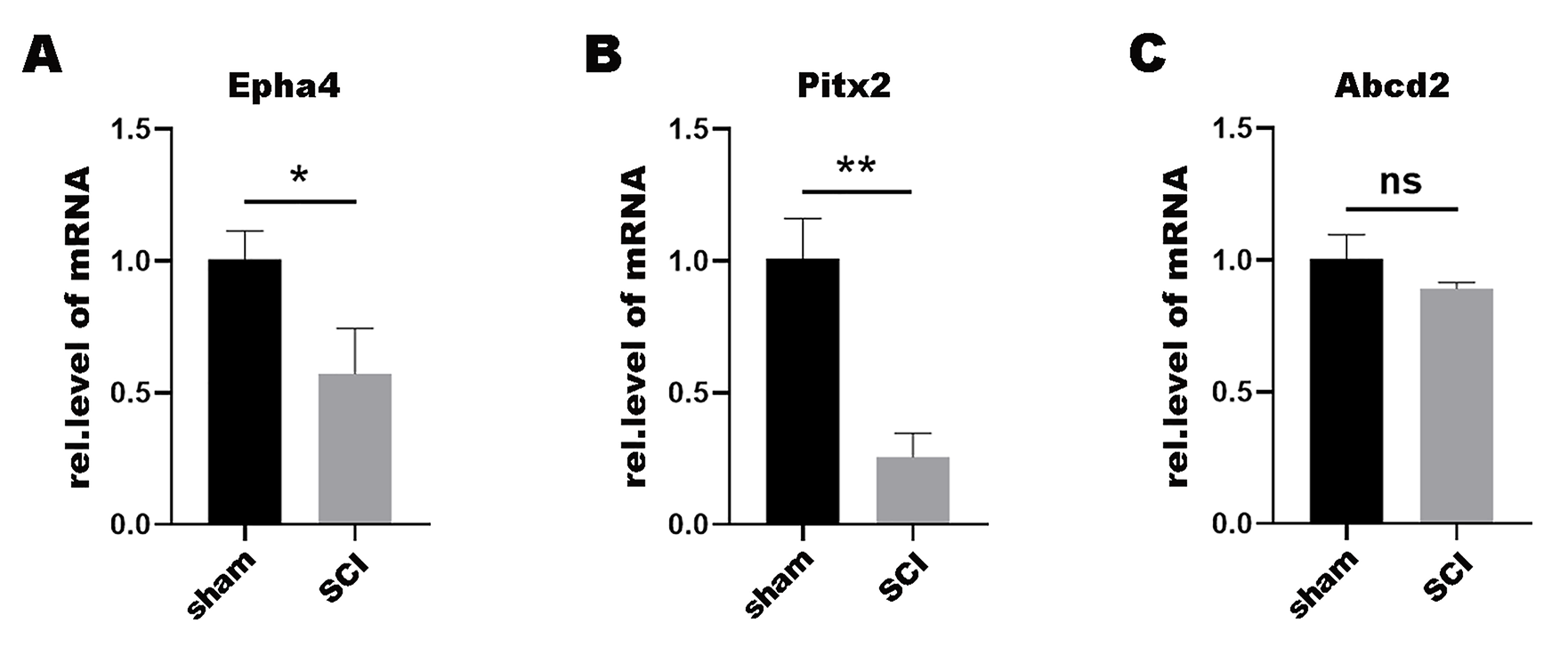

Supplement: Supplementary file 1 — Supplementary figures and tables. [file ijbsv17p2795s1.zip › Supplementary materials/zyn sfig2.tif]

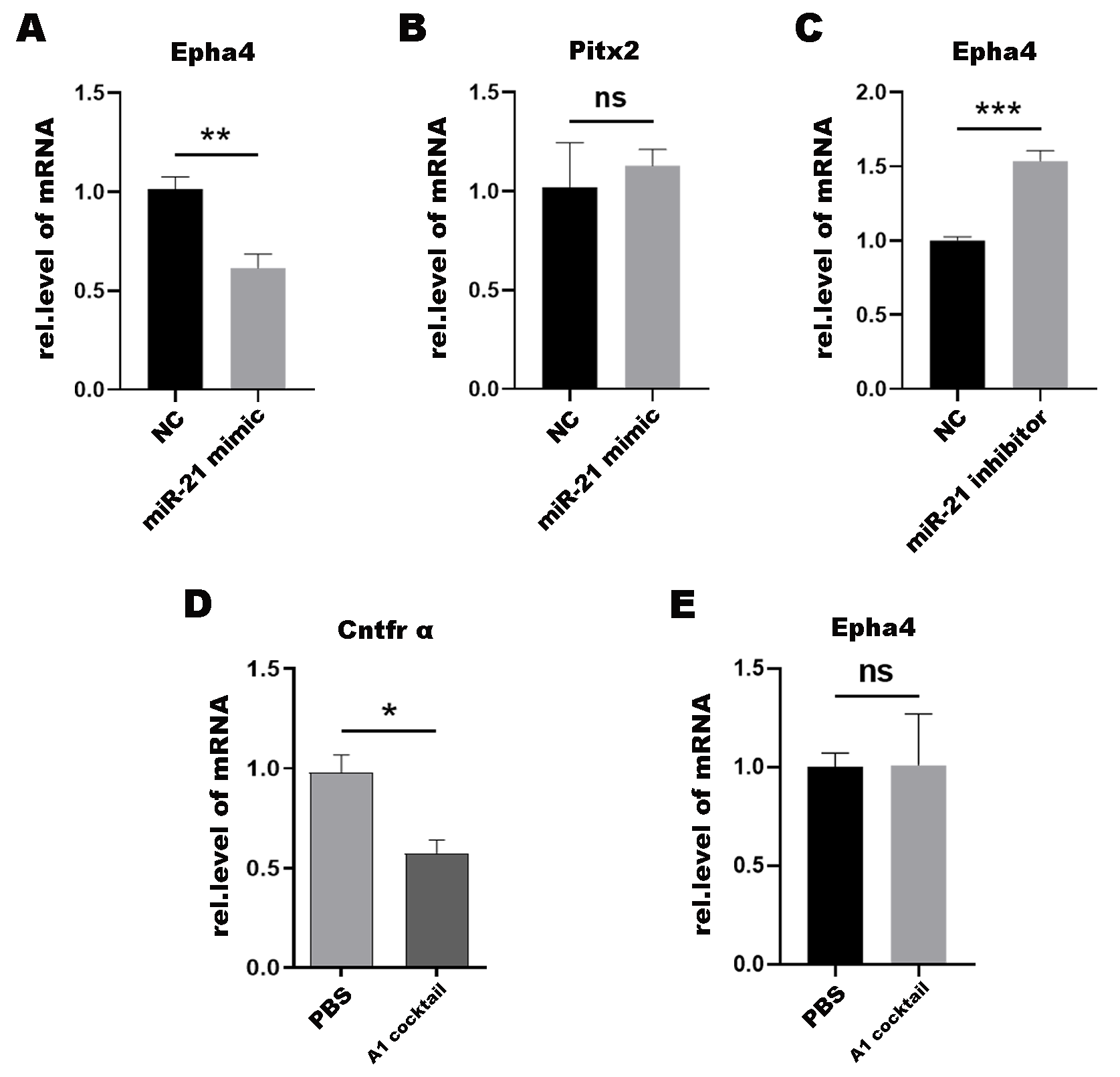

Supplement: Supplementary file 1 — Supplementary figures and tables. [file ijbsv17p2795s1.zip › Supplementary materials/zyn sfig3.tif]
